# Mass Cytometry Identifies Distinct Subsets of Regulatory T Cells and Natural Killer Cells Associated With High Risk for Type 1 Diabetes

**DOI:** 10.3389/fimmu.2019.00982

**Published:** 2019-05-03

**Authors:** Hugo Barcenilla, Linda Åkerman, Mikael Pihl, Johnny Ludvigsson, Rosaura Casas

**Affiliations:** ^1^Division of Pediatrics, Department of Clinical and Experimental Medicine, Faculty of Medicine and Health Sciences, Linköping University, Linköping, Sweden; ^2^Core Facility, Flow Cytometry Unit, Linköping University, Linköping, Sweden; ^3^Crown Princess Victoria Children's Hospital, Region Östergötland, Linköping, Sweden

**Keywords:** type 1 diabetes (T1D), high-risk for T1D, autoantibody-positive children, mass cytometry (CyTOF), regulatory T cells, NK cells

## Abstract

Type 1 diabetes (T1D) is characterized by autoimmune destruction of insulin producing β-cells. The time from onset of islet autoimmunity to manifest clinical disease can vary widely in length, and it is fairly uncharacterized both clinically and immunologically. In the current study, peripheral blood mononuclear cells from autoantibody-positive children with high risk for T1D, and from age-matched healthy individuals, were analyzed by mass cytometry using a panel of 32 antibodies. Surface markers were chosen to identify multiple cell types including T, B, NK, monocytes, and DC, and antibodies specific for identification of differentiation, activation and functional markers were also included in the panel. By applying dimensional reduction and computational unsupervised clustering approaches, we delineated in an unbiased fashion 132 phenotypically distinct subsets within the major immune cell populations. We were able to identify an effector memory Treg subset expressing HLA-DR, CCR4, CCR6, CXCR3, and GATA3 that was increased in the high-risk group. In addition, two subsets of NK cells defined by CD16^+^ CD8^+^ CXCR3^+^ and CD16^+^ CD8^+^ CXCR3^+^ CD11c^+^ were also higher in the same subjects. High-risk individuals did not show impaired glucose tolerance at the time of sampling, suggesting that the changes observed were not the result of metabolic imbalance, and might be potential biomarkers predictive of T1D.

## Introduction

Type 1 diabetes (T1D) is characterized by autoimmune destruction of insulin producing β-cells. The time from onset of islet autoimmunity to manifest clinical disease can vary widely in length (1) but the pre-diabetic period is fairly uncharacterized both clinically and immunologically. Since several genes are involved in disease susceptibility, one common approach to identify individuals at risk for T1D is to assess the genetic risk (2). However, HLA-typing is a poor prognostic tool considering that 10–30% of the general Caucasian population, depending on criteria, carry HLA alleles that confer susceptibility for T1D (3). More accurate disease prediction can be achieved by assessment of autoantibodies directed toward β-cell antigens like the 65 kD isoform of glutamic acid decarboxylase (GAD65) (4), protein tyrosine phosphatase-like islet antigen (IA)-2, insulin (IA) (5) and zinc transporter (ZnT)-8 (6). Presence of autoantibodies in serum indicates ongoing islet autoimmunity, and the risk for development of overt diabetes is especially high when multiple autoantibodies are present (7, 8). Increasing knowledge on the period preceding disease onset indicates a heterogeneous disease process, best characterized by the combination of disease-related risk genes, autoantibody profile and glucose homeostasis. However, much of what is known about the pre-diabetic period comes from studies with first- or second-degree relatives of T1D patients.

T1D is often viewed as a T cell-driven autoimmune disease, particularly for the more prevalent and aggressive type of T1D that develops in children and adolescents vs. adults (9–13). Immune system deviations such as islet antigen specific autoreactive CD8^+^ and CD4^+^ T cells have been detected in peripheral blood of T1D patients, in some at-risk individuals and occasionally in healthy individuals (14). Although T cells are largely considered to be responsible for β-cell destruction in T1D, increasing evidence points toward a role for B cells in the disease pathogenesis (15).

The recent introduction of mass cytometry has transformed the understanding of the complexity and function of the immune system (16, 17). Mass cytometry overcomes the main limitation of conventional fluorescence-based flow cytometry, expanding the number of immune features that can be simultaneously measured at the single-cell level. The high-dimensional datasets generated by mass cytometry are not well-suited for manual gating strategies. Thus, novel computational data analysis approaches have been developed (18–21), changing the way originated data are handled (22). In the current study, we took advantage of the increased parameterization offered by mass cytometry to simultaneously interrogate immune subsets across all major lineages in human PBMC from children with high risk for T1D, positive for multiple autoantibody, and from age-matched healthy individuals.

## Materials and Methods

### Subject Characteristics

Children with high risk for T1D were identified, regardless of genetic background, through autoantibody screening in the general population in the All Babies in Southeast Sweden (ABIS) birth-cohort study. The ABIS study consisted of 17,055 children from the general Swedish population (23) and provided the opportunity to identify and prospectively follow children with increased risk of T1D. Children positive for multiple diabetes-related autoantibodies on at least two screening occasions (at age 1, 2.5–3, 5–6, 8, and 11–12) were considered at high risk for T1D development. The high-risk individuals participated in a 2 year prospective follow-up study involving blood sampling every 6 months for measurement of fasting blood glucose, autoantibodies, C-peptide, HbA1c, and for HLA-genotyping, and an oral glucose tolerance test (OGTT) once every year (24).The risk individuals included in the present study (*n* = 9, Table 1) all developed T1D during or after the follow-up period, and the samples for the study were drawn on the last visit before clinical disease onset (average 13 months pre-onset, range 5–26 months). The healthy age-matched controls (*n* = 9, Table 1) were also selected among ABIS participants. They were all negative for islet autoantibodies, had no diabetes type 1 or 2, or any autoimmune disorder, no asthma, eczema or allergies, and no first-degree relatives with diabetes or autoimmune disorders.

**Table 1:** Characteristics of all individuals included in the study at the time of sampling.

	**Gender**	**Age at sampling**	**Time between sample and onset (months)**	**Fasting C-peptide (nmol/l)**	**HbA1c (%)**	**Fasting glucose (nmol/l)**	**120 min OGTT (nmol/l)**	**GADA (units/ml)**	**IA2A (units/ml)**	**IAA (units/ml)**	**ZnT8Atrp (units/ml)**	**ZnT8Aarg (units/ml)**	**ZnT8Aglt (units/ml)**
**HIGH-RISK**													
	M	12	9	0.6	4.4	5.4	9.1	6.350	1.624	37.3	772.4	1.158	353.9
	F	12	6	0.85	4.7	5.2	6.1	6.400	neg	19.5	1.808	788	125.9
	M	12	8	0.52	4.6	6.1	8.4	5.626	neg	neg	neg	319.7	neg
	M	13	10	0.44	5	5.4	6.1	113.4	2.920	57	636.8	3915	1.209
	M	11	15	0.39	4.5	5.1	na	78.3	7.200	7	2.418	neg	neg
	F	12	5	0.42	4.6	5.7	na	253.8	neg	neg	neg	neg	neg
	F	11	6	0.39	4.6	5.4	na	11.800	13.5	neg	neg	neg	neg
	M	13	16	0.84	4.4	5.4	6.1	1.231	1.970	39.1	neg	2.225	neg
	M	11	25	0.6	4.5	5.2	7.9	420.4	664.5	neg	neg	1.332	neg
**CONTROLS**													
	Male	12	–	0.69	na	na	na	neg	neg	neg	neg	neg	neg
	Male	12	–	0.35	na	na	na	neg	neg	neg	neg	neg	neg
	Male	12	–	0.55	na	na	na	neg	neg	neg	neg	neg	neg
	Male	12	–	0.39	na	na	na	neg	neg	neg	neg	neg	neg
	Male	12	–	0.32	na	na	na	neg	neg	neg	neg	neg	neg
	Male	12	–	0.35	na	na	na	neg	neg	neg	neg	neg	neg
	Male	12	–	0.34	na	na	na	neg	neg	neg	neg	neg	neg
	Female	11.5	–	0.41	na	na	na	neg	neg	neg	neg	neg	neg
	Female	10	–	0.54	na	na	na	neg	neg	neg	neg	neg	neg

*HbA1c, glycated hemoglobin A1c; OGTT, oral glucose tolerance test; Autoantibodies to glutamic acid decarboxylase 65 (GADA), protein tyrosine phosphatase-like islet antigen (IA2A), insulin (IAA) and zinc transporter 8 (ZnT8A). F, Female; M, Male; na, not available; neg, negative (values below the cut-off for autoantibody positivity)*.

### Sample Preparation

Peripheral blood mononuclear cells (PBMC) were separated by density gradient centrifugation in Leucosep tubes (Greiner Bio One) according to manufacturer's instructions and cryopreserved in medium containing 10% DMSO/FCS.

### Cell Staining

The antibody panel, stimulation conditions and intracellular staining used for mass cytometry in this study were optimized and validated by flow cytometry using BD FACSAria III. Details of antibodies used are listed in Supplementary Table 1. When indicated, purified carrier-free antibodies were purchased from the companies listed and conjugated with metal isotopes using Maxpar antibody labeling kit (Fluidigm) according to the manufacturer's instructions.

Cryopreserved PBMC were thawed and washed with pre-warmed RPMI 1640 medium supplemented with 10% FCS, and rested overnight at 37°C. Then, cells were washed, and samples were split into two tubes and then stained at 37°C with anti-CD4-^144^Nd. The addition of this antibody prior to stimulation improves the staining of anti-CD4 upon stimulation, as shown previously (25). Cells were left untreated or stimulated for 4 h with 100 ng/ml phorbol-12-myristate-13-acetate (PMA) and 1 μg/ml ionomycin in the presence of 3 μg/ml Brefeldin A (eBioscience) and 2 μM monensin (eBioscience).

After incubation, cells were washed twice in PBS, and then stained for dead cell discrimination with 2.5 μM cisplatin (Fluidigm) for 5 min at room temperature and quenched with RPMI+10% FCS. Cells were then washed in cell staining buffer (CSM, Fluidigm), and stained for surface markers with a cocktail of metal-conjugated antibodies (Supplementary Table 1) for 30 min at 4°C. Cells were washed twice in CSM and resuspended in FOXP3 fixation/permeabilization buffer (eBioscience). After 40 min, samples were washed twice with permeabilization buffer (eBioscience) and stained with the intracellular antibody cocktail (Supplementary Table 1). After 45 min, cells were washed twice with permeabilization buffer, once with CSM, and then fixed in PBS with 2% paraformaldehyde (PFA) overnight at 4°C.

### Sample Barcoding and Data Acquisition

To reduce data collection variability, samples were processed in batches of 5, including similar number of samples from each group, and barcoded with combinations of three unique palladium isotopes (26). Barcoding was performed after antibody staining, as PFA-based fixation used during barcoding resulted in drastic reduction in transcription factor staining when used before the FOXP3 fixation/permeabilization buffer (Supplementary Figure 1).

Cells were barcoded using Cell-ID 20-plex Pd-barcoding kit (Fluidigm) according to the manufacturer's instructions. Briefly, cells were washed twice with Maxpar barcode perm buffer (Fluidigm), and a different barcode set was added to each sample for 30 min at room temperature. After washing the samples twice with CSM, all samples were combined into one tube. Finally, cells were stained with 125 nM Ir191/193 DNA intercalator (Cell-ID Intercalator-Ir, Fluidigm) for 20 min, washed in Di water, filtered through a 35 μm nylon mesh and resuspended to 0.5 × 10^6^ cells/ml with 0.1% EQ four element calibration beads (Fludigm). Data acquisition was done with a CyTOF 2 mass cytometer (Fluidigm) at an event rate of 300–500 cells/s. After data acquisition,.fcs files were concatenated, normalized using mass bead signal (27) and debarcoded using the CyTOF 2 software prior to analysis.

### Regulatory T Cell Sorting and Expansion

PBMC were stained with Pacific Blue conjugated anti-CD4, FITC-conjugated anti-CD127, and APC-conjugated anti-CD25, and regulatory T cells (Tregs) were sorted based on the expression of CD4^+^CD25^hi^CD127^lo^. After sorting (BD FACSAria III) cells were pelleted by centrifugation at 400 g for 10 min, resuspended in AIM-V 10% human serum (HS) and allowed to rest for 2 h at 37°C, 5% CO_2_ before expansion. Aliquots of sorted cells were re-acquired to assess purity, showing an average of contamination of 0.1%. Sorted Treg were distributed at 4 × 10^4^ cells per well into round-bottom 96-well plates containing 125 μl AIM-V 10% HS, and then stimulated with anti-CD3/CD28 Dynabeads (Life Technologies) at a 1:1 bead-to-cell ratio. Culture volume was doubled the following day and 300 U/ml of recombinant human IL-2 (R&D Systems) was added. Cells were washed and supplemented with fresh IL-2 every 2 days and re-stimulated as above on the ninth day of culture.

### Suppression Assays

Suppression assays were performed by flow cytometry as described previously (28). Briefly, carboxyfluorescein succinimidyl ester (CFSE)-labeled Teff were cultured in the presence of either Tregs or control cells stained with CellTrace Violet at different ratios. Cultures were stimulated with anti-CD3/CD28 coated beads at a ratio of 1:20 (bead:cell). Teff proliferation was calculated as Division Index after 72 h of culture. For accurate cell counting in each cell division, equal amounts of reference beads were added immediately prior to acquisition.

### Data Analysis and Statistics

**Figure 1:**
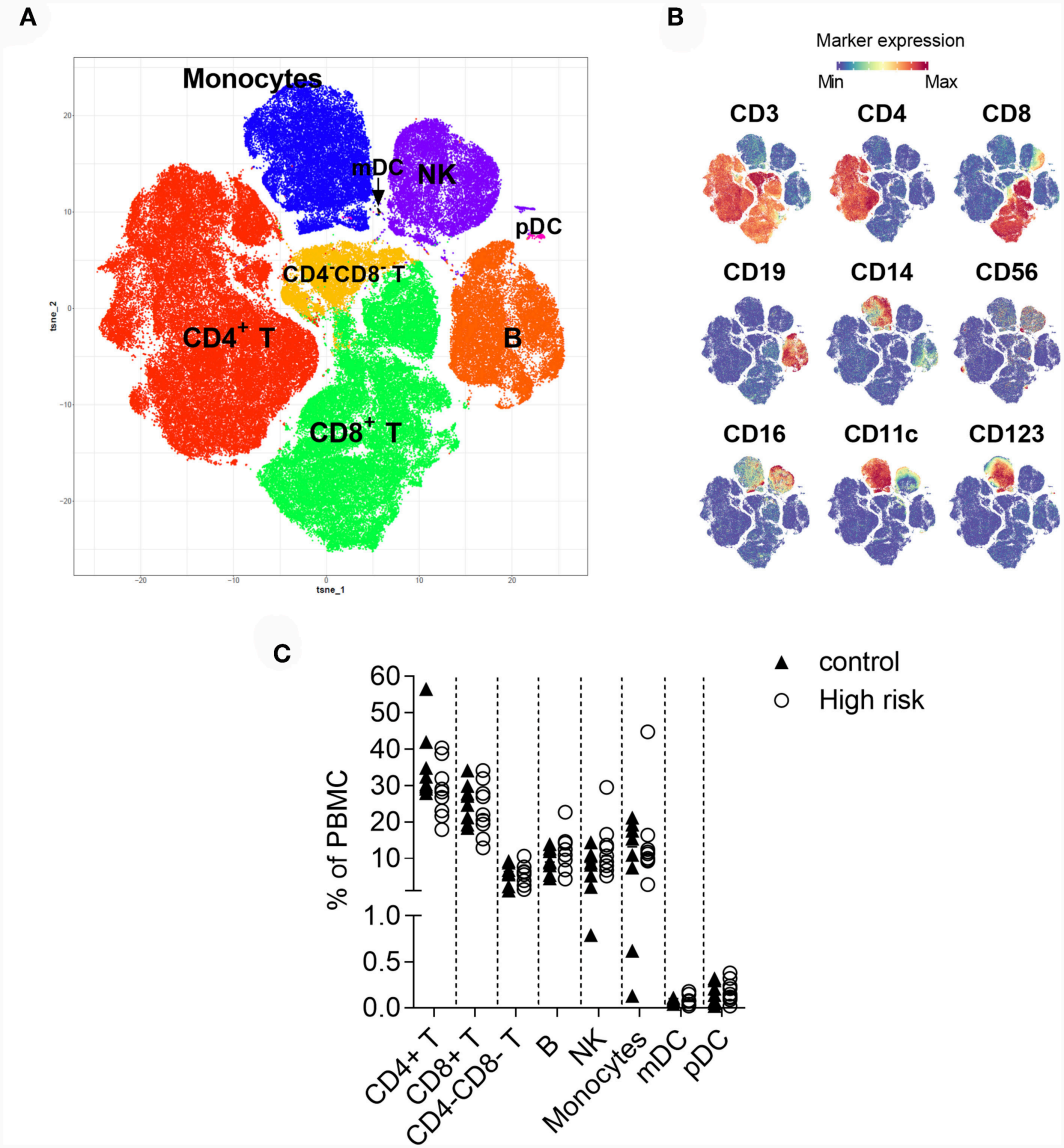
t-Distributed Stochastic Neighbor Embedding (t-SNE) analysis of CD45^+^ cells. **(A)** Two-dimensional map where cells (dots) are plotted according to their expression of all markers. **(B)** Immune cell populations are annotated based on the intensity of expression of lineage antigens. CD4^+^ (CD3^+^CD4^+^), CD8^+^ (CD3^+^CD8^+^), CD4^−^CD8^−^ (CD3^+^CD4^−^CD8^−^) T cells, B cells (CD19^+^), monocytes (CD14^+^), NK cells (lineage^−^, CD56^+^), mDC (lineage^−^CD11c^+^CD123^−^), pDC (lineage^−^, CD11c^−^CD123^+^). The arrow indicates the position of the mDC. **(C)** Percentage of CD4^+^ T, CD8^+^ T, CD4^−^CD8^−^ T, B and NK cells, monocytes, and myeloid and plasmacytoid dendritic cells within peripheral mononuclear cells (PBMC) in individuals with high-risk for type 1 diabetes (*n* = 9, white circles) and controls (*n* = 9, black triangles). Dots represent individual samples. Differences between groups were tested using Mann-Whitney U-test. No significant differences were found.

Manual gating of.fcs files was performed using Cytobank (29). Calibration beads and cell aggregates were first excluded by gating on 140Ce events and using 193Ir/191Ir DNA signal vs. event length, as previously described (30). For the t-Distributed Stochastic Neighbor Embedding (t-SNE) analysis shown in Figure 1, live (^195^Pt^−^) CD45^+^ cells from each sample were gated, exported and analyzed with version 1.10.4 of the “cytofkit” R package (31), using cytofAsinh transformation and t-SNE visualization. Only lineage markers (CD markers) were used for this analysis. The 2-dimensional t-SNE maps and the marker expression heatmaps were visualized using R package “Shiny,” and plots were colored according to the expression of lineage markers. For subset identification, exported events from each population were imported into cytofkit and analyzed with Phenograph with the following settings: merge: ceil, transformation: cytofAsinh, cluster: RPhenograph, visualization: t-SNE. Resulting cluster.fcs files were imported into Cytobank for further examination and detection of spurious clusters. All the markers (CD markers, chemokines, transcription factors and cytokines) were used for the generation of t-SNE maps for each population. Median arcsinh expression heatmaps were created in Cytobank. For cytokine expression, differential expression between un-stimulated and stimulated samples was calculated. All subsets generated with Phenograph were analyzed for statistical significance between groups in GraphPad Prism v7 using a Mann-Whitney U-Test. *p* < 0.05 was considered significant.

Citrus algorithm analysis was performed using version 0.08 in R. Live (195Pt^−^) CD45^+^ cells were imported and down-sampled to 10,000 events per file. The Nearest Shrunken Centroid association model was applied with 0.5% minimum cluster size and 5 cross-validation fold. Model error rate was used to evaluate model fit.

## Results

We developed a panel of 33 metal-labeled monoclonal antibodies for the high-dimensional analysis of multiple cell types within PBMC. Surface markers were chosen to identify T and B lymphocytes, natural killers (NK), monocytes and dendritic cells (DC), and antibodies specific for differentiation, activation and function were also included in the panel (Supplementary Table 1). Samples from individuals with high risk for T1D (*n* = 9) and healthy controls (*n* = 9) were included in this study.

First, to visualize the cellular heterogeneity of PBMC, t-SNE analysis (32) was applied to similar number of live single CD45^+^ cells from all the individuals. This approach generates a two-dimensional map where similar cells are placed at adjacent points, while cells with different characteristics are separated in space (Figure 1A). Eight major immune lineages corresponding to CD4 T cells, CD8 T cells, double negative (CD4^−^CD8^−^) T cells, B cells, NK cells, monocytes, myeloid dendritic cells (mDC) and plasmacytoid dendritic cells (pDC) were defined based on lineage marker expression (Figure 1B). The distribution of these major populations was similar in the samples from the high-risk individuals and the healthy control group (Figure 1C). Cell frequencies obtained through t-SNE analysis were confirmed by manual gating (Supplementary Figure 2).

### High Dimensional Analysis Reveals Heterogenicity Within the PBMC Cells

**Figure 2:**
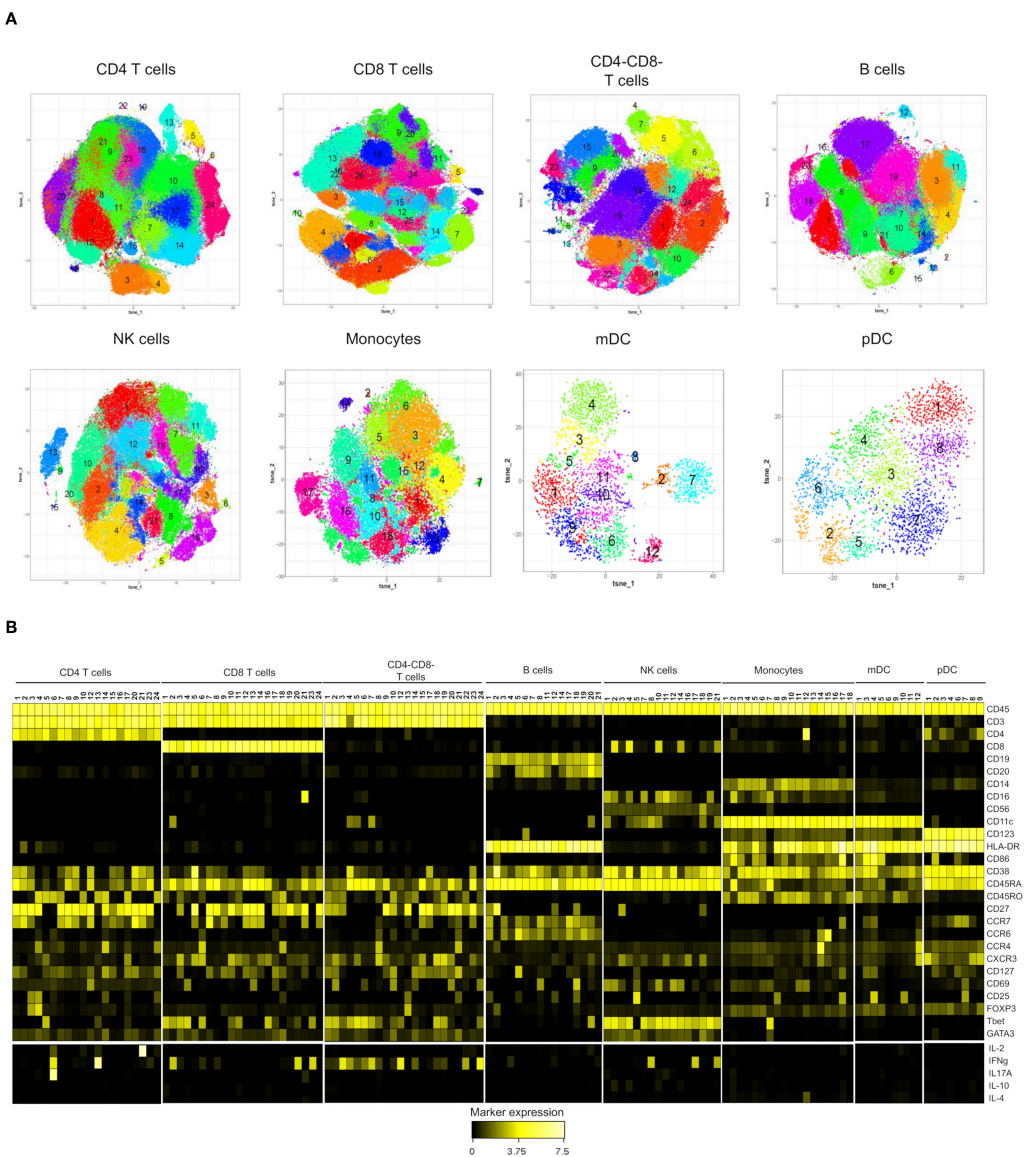
Subset identification within the major immune populations in peripheral mononuclear cells from high-risk (*n* = 9) and control individuals (*n* = 9). **(A)** Phenotypically distinct subsets were identified with t-SNE combined with Phenograph analysis of each lineage population (colored areas) **(B)** Heatmap showing median expression of the markers expressed by the cell subsets identified in **(A)**. Subsets of CD4+ (11,18,19,22,25), CD8+ (15, 20, and 22), CD4-CD8- (11,16), B (9,10,13,15,16) NK (6,9,13,15,20), mDC (2,7,8) and pDC (5) were excluded from the heatmap. Cytokine (IL-2, IFNγ, IL-17A, IL-10, and IL-4) expression is shown as subtraction of the expression on un-stimulated PBMC from samples stimulated with PMA+ ionomycin. Values were transformed using arcsinh function in a cofactor of 5.

Based on the expression of all analysis parameters, we assessed next the complexity of each major population individually. Automatic subset identification was done with t-SNE and the Phenograph clustering algorithm. Cluster analysis identified a total of 154 phenotypically distinct subsets (Figure 2A). Among them, 22 were excluded as they expressed markers that were not specific for any lineage population, and/or contained low number of cells. The exclusion was decided after manual analysis of the data (.fcs files) extracted from each cluster.

The phenotypic characteristics of the clusters, ordered according to their lineage and the median expression of every marker, are displayed in a heatmap (Figure 2B).

Using the Phenograph algorithm, we were able to identify a relatively large number of clusters within the T cell populations, likely explained by a greater number of markers that are differentially expressed on T cells in our panel. For instance, the expression of activation markers, chemokine receptors, transcription factors and cytokines revealed a high degree of heterogeneity within CD4 T cells, and 20 distinct subsets across naïve (CD45RA^+^CD45RO^−^CCR7^+^, 11 clusters), central memory (CD45RA^−^CD45RO^+^CCR7^+^, 4 clusters), and effector memory (CD45RA^−^CD45RO^+^CCR7^−^, 5 clusters) subpopulations were identified (Figure 2B and Supplementary Table 2). Among the memory clusters, we identified classical subsets like CXCR3^+^CCR4^−^Tbet^+^ (Th1 cells) and CXCR3^−^CCR4^+^GATA3^+^ (Th2 cells). The clustering algorithm also revealed unexpected distribution of the Th2 and Th17-associated chemokine receptors, CCR4 and CCR6, that were co-expressed in several subsets. We also detected a Th2-like Treg population co-expressing FOXP3 and GATA3. In addition, a Th17 cluster expressing predominantly IL-17 also expressed lower levels of IL-2 and IFNγ upon PMA stimulation. The analysis of CD8^+^ T cells identified 22 phenotypically distinct clusters (Figure 2B and Supplementary Table 3). Among them, 9 clusters were CD8^+^ naïve T cells (CD45RA^+^CD45RO^−^CCR7^+^) that were distinguished by different expression of CD127, CXCR3, HLA-DR and CD69. Most of the cells with central memory phenotype (CD45RA-CCR7+) were spread within effector CD8+ T cells (CD45RA-CCR7- and CD45RA+CCR7-) that expressed Tbet and were differentiated by the expression of various activation and/or trafficking markers and the production of IFNγ. However, we identified a defined subset of central memory CD8+ T cells characterized by the expression of CCR4, CD25, and GATA3. It was interesting that despite their Tc2 phenotype, this cluster did not express IL-4.

Clustering of double negative CD4^−^CD8^−^ cells within the CD3^+^ population identified 22 cell subsets (Figure 2B and Supplementary Table 4), that were predominantly memory/effector CCR7^−^ cells, all of them displaying a Th1-like phenotype (CXCR3^+^Tbet^+^). After PMA stimulation, 4 subsets expressed IFNγ at higher levels than those observed in CD8^+^ T cells.

The cluster analysis of B cells, NK cells, monocytes, mDC and pDC showed a smaller number of phenotypically distinct clusters (Figure 2B and Supplementary Tables 5–9). However, very interesting populations like the intriguing Tbet^+^CD11c^+^ B cell and the CD8^+^CD11c^+^ NK cell subsets were observed. One interesting finding within NK cells was that GATA3 expression was lost only in the 3 subsets producing IFNγ upon stimulation.

### Treg and NK Cell Subsets Increased in High-Risk Individuals

**Figure 3:**
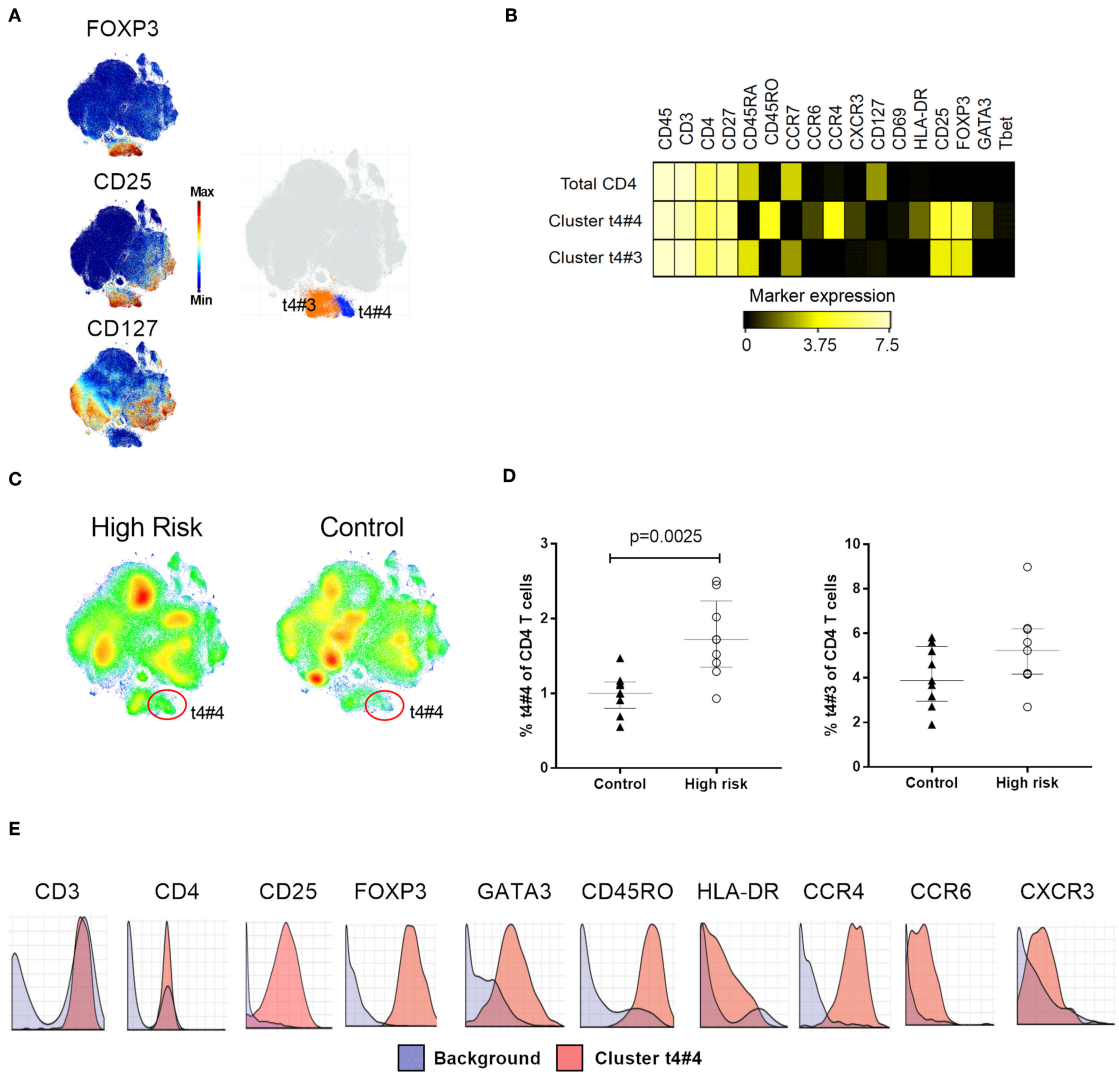
Regulatory T cell phenotyping. **(A)** Clustering analysis on CD4^+^ T cells, high expression of FOXP3 and CD25, and absence of CD127 delineated a Treg area with two Treg subsets, t4#3 and t4#4, highlighted as orange and blue, respectively on the t-SNE map. **(B)** Median expression of relevant phenotype markers that identified Treg subsets t4#3 and t4#4. **(C)** t-SNE plots illustrating cellular density in individuals with high risk for type 1 diabetes and controls. **(D)** Percentage of t4#3 and t4#4 subset within total CD4 T cells in high-risk individuals (white circles) and controls (black triangles). **(E)** Citrus-generated histograms showing distribution of phenotype markers expressed by cluster t4#4 (red) in relation to the background expression (blue). Background was determined by all the cells that do not belong to clusters significantly different between the groups, i.e., whole PBMC. Dots represent individual samples. Error bars show median ± interquartile range. Median expression values were transformed using arcsinh function in a cofactor of 5. *p*-values for differences between groups were determined using Mann-Whitney U-test. *p* < 0.05 was considered significant.

To search for differences between individuals with high risk for T1D and healthy controls, we compared the profile of all the t-SNE maps generated during clustering analysis, stratified for the two groups. Using the combination of Phenograph and t-SNE analysis, we found that although the maps for each major cell population were similar in shape and number of clusters, the groups displayed distinct cellular densities, showing subsets that appeared to be differentially abundant. The statistical analysis of the frequencies of all cell subsets revealed differences only in clusters within CD4+ T and NK cells, that were more abundant in individuals at high risk for T1D. Two of these subsets were located in a clearly delimited area within CD4+ cells and had a Treg phenotype, (cluster ID: t4#3 and t4#4), defined by the expression of FOXP3 and CD25 and the absence of CD127 (Figure 3A). The subset t4#4 displayed an effector memory phenotype (CD45RA^−^CCR7^−^CD45RO^+^) and expressed the transcription factor GATA3 together with HLA-DR, CCR4 and CCR6. The other subset, t4#3, had a naïve phenotype (CD45RA^+^CCR7^+^CD45RO^−^), and cells in this cluster did not express GATA3, CCR4, CCR6, or HLA-DR (Figure 3B). Higher cell density of the subset t4#4 in high-risk individuals was clearly observed in the density maps generated by t-SNE (Figure 3C). Moreover, this subset was significantly more abundant in the high-risk group (*p* = 0.0025, Figure 3D).

**Figure 4:**
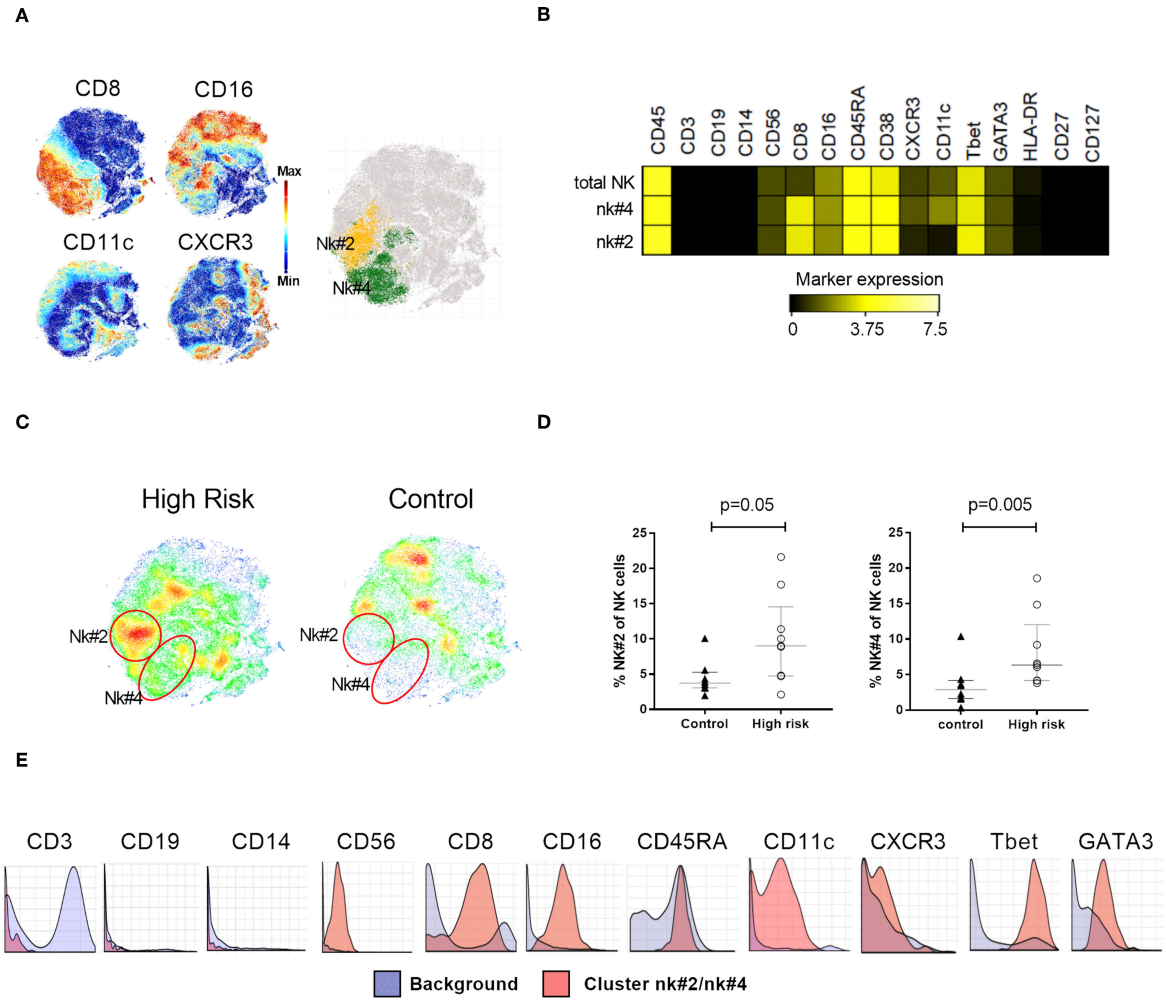
NK cell phenotyping. **(A)** Clustering analysis on NK cells expressing CD8, CD16, CD11c, and CXCR3 characterized two NK subsets, nk#2 (yellow) and nk#4 (green), highlighted on the t-SNE map **(B)** Median expression of relevant markers used for the identification of NK subsets nk#2 and t4#4. **(C)** t-SNE plots illustrating cellular density in individuals with high risk for type 1 diabetes and controls. **(D)** Percentage of nk#4 and nk#2 subsets within total NK cells in high-risk individuals (*n* = 9, white circles) and controls (*n* = 9, black triangles). **(E)** Citrus-generated histograms showing marker distribution in cluster nk#2/nk#4 (red) in relation to background expression (blue). Background was determined by all the cells that don not belong to the clusters significantly different between the groups, i.e., whole PBMC. Dots represent individual samples. Error bars show median ± interquartile range. Median expression values were transformed using arcsinh function in a cofactor of 5. *p*-values for differences between groups were determined using Mann-Whitney U-test. *p* < 0.05 was considered significant.

Our analysis also identified two subsets within the NK cell compartment (cluster ID: nk#4 and nk#2), that were clustered in an area defined by the expression of CD8 and low expression of CD16 and CXCR3 (Figure 4A). These two clusters were distinguished from each other by the expression of CD11c, present in the nk#4 subset but absent in nk#2 (Figure 4B). These populations were both more abundant in high-risk individuals than in the control group (nk#4: *p* = 0.005; nk#2: *p* = 0.05, Figures 4C,D).

Cell density t-SNE maps of both CD4^+^ T and NK cells presented additional areas of different densities between high-risk and control groups. Subsets included in these areas showed high inter-individual variation and were not statistical different between the groups (Supplementary Figure 3).

### Treg and NK Subsets Were Also Identified by Citrus Algorithm and Manual Gating

To assess the validity of the findings detected with t-SNE analysis combined with Phenograph, we applied next the Citrus algorithm (33). This analysis merges cells across all samples, performs hierarchical clustering, and then selects the features best distinguishing different experimental groups. We applied the nearest shrunken centroid predictive model on CD45^+^ live cells, and identified two clusters, with identical phenotypic characteristics as those detected by Phenograph. One of them was the memory-like Treg subset expressing GATA3, CCR4, HLA-DR, and CCR6 (cluster t4#4, Figure 3E) and the other was the NK cell subset expressing CD8, CD16, CXCR3, and CD11c (cluster nk#2/nk#4, Figure 4E), both more abundant in the high-risk group than in the control individuals.

We further confirmed the presence of Treg and NK cell subsets increased in high-risk individuals by manual analysis of the data based on the expression of the markers identified by Phenograph and citrus (Supplementary Figure 4).

### Cytokine Secretion by Treg and NK Subsets

We next analyzed the intracellular cytokine profile of the Treg and NK cell subsets that differed between high-risk and healthy children. Stimulation with PMA and ionomycin did not affect the identification of the Treg subsets (t#3 and t#4). CD4+ T cells producing IFNγ, IL-2, IL-4, and IL-17 were clustered outside the Treg area (Supplementary Figure 5A and Supplementary Table 2). Although IL-10 secreting cells did not form a cluster within stimulated T cells, manual gating revealed small number of scattered IL-10+ Tregs, equally disseminated within the two Treg subsets. The frequency of this IL-10+ Tregs was not significantly different between the groups (Supplementary Figure 5B).

Cytokine secretion in NK cells was limited to IFNγ. Producing cells clustered in 2 subsets of CD8– cells and a smaller subset of CD8+ cells that were similar in high-risk and control individuals (Supplementary Figure 5C and Supplementary Table 6). The expression of CD16, CXCR3 and GATA3 was down-regulated in stimulated NK cells, and thus we were not able to identify the NK cell subsets nk#2 and nk#4 by Phenograph as independent clusters. Manual analysis confirmed that IFNγ production was mainly located in CD8-CD11c+ and CD8-CD11c– NK cells. The proportion of IFNγ+ cells sharing phenotypic features with the subsets nk#2 and nk#4 (CD8+CD11c– and CD8+CD11c+cells, respectively) was not significantly different between the high-risk and control groups (Supplementary Figure 5D).

### Treg Expansion and Suppressive Capacity

**Figure 5:**
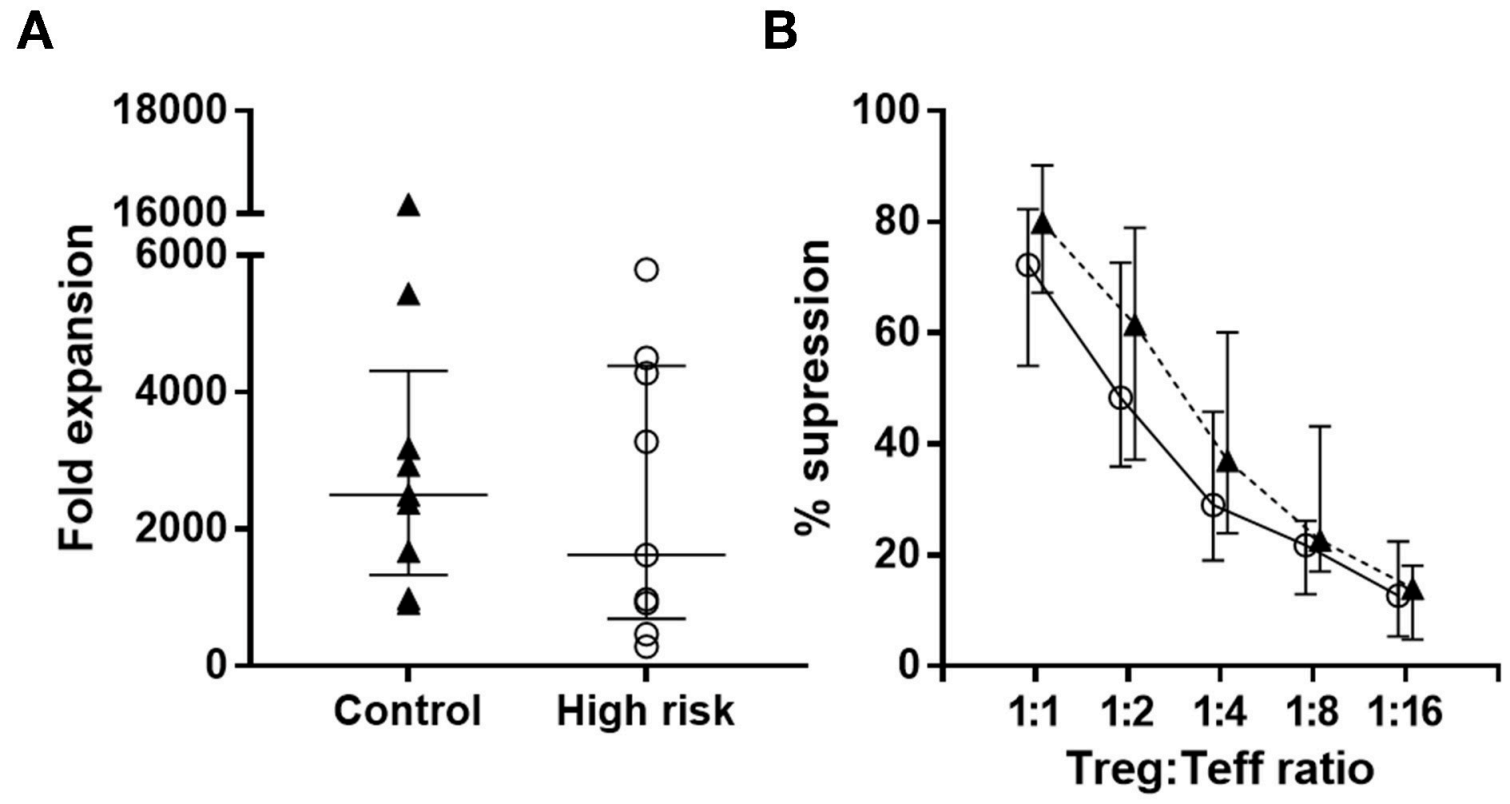
Treg expansion and function. **(A)** Fold expansion of Treg from high-risk individuals (*n* = 9, white circles) and controls (*n* = 9, black triangles) after 2 weeks expansion. Dots represent individual samples. Error bars show median ± interquartile range **(B)** Suppression exerted by Tregs on autologous Teff from high-risk individuals (white circles) and controls (black triangles) at different Treg/Teff ratios. Data are presented as median ± interquartile range. *p*-values for differences between groups were determined using Mann-Whitney U-test. No significant differences were found.

We next set out to determine whether Treg from high-risk and healthy individuals were able to suppress Teff to a similar extent. Sorted CD4^+^ CD25^hi^CD127^lo^ Tregs were expanded in presence of anti-CD3/CD28 antibodies and high-dose IL-2 for 2 weeks. We observed that fold-increase of the Tregs from both groups was similar (Figure 5A).

To assess the suppressive capacity of expanded Tregs, we analyzed the proliferation of autologous CFSE-labeled Teff in the presence of Tregs at different Treg/Teff ratios. Results from the suppression experiments showed that the suppressive activity of Tregs from both groups was similar (Figure 5B).

## Discussion

In this study, we performed high-dimensional single-cell profiling of human PBMC from children at high risk for T1D and age-matched healthy individuals. We took advantage of the increased parameterization offered by mass cytometry to perform a comprehensive profile of the samples. By applying dimensional reduction and computational unsupervised clustering approaches we delineated 132 phenotypically distinct subsets within the major immune cell populations in an unbiased fashion. We were able to identify an effector memory Treg subset expressing HLA-DR, CCR4, CCR6, CXCR3, and GATA3 that was increased in the high-risk group. In addition, two subsets of NK cells defined by CD16^+^ CD8^+^ CXCR3^+^ and CD16^+^ CD8^+^ CXCR3^+^ CD11c^+^ were also higher in the same samples.

The role of Tregs in T1D has been studied extensively over the last years, and there has been a consensus that the frequency of Tregs in T1D is not altered but rather their function seems to be impaired (34–37). Studies including individuals at risk to develop T1D identified by the detection of multiple autoantibodies are limited. It has recently been shown that the proportion of peripheral blood Treg in autoantibody positive high-risk individuals was not altered (38), but Treg phenotyping in the study was limited to the expression of CD4, CD25, and FOXP3. We interrogated the human PBMC compartment with an extensive antibody panel, including differential expression of transcription factors, chemokine receptors and activation markers. Using mass cytometry, we were able to combine several markers that otherwise are not commonly analyzed together. In line with a previous study performing deep phenotyping of PBMC with the same methodology (39), we identified two well-defined clusters within the CD25^hi^FOXP3^+^CD127^−^ area of CD4^+^ T cells, displaying memory and naïve phenotype, respectively. We observed that the majority of Tregs corresponded to the cluster with naïve phenotype, and they were similarly abundant in high-risk and control individuals. Interestingly, the Treg subset increased in the high-risk group consisted of effector memory cells, and they were predominantly CCR4^+^ CCR6^+^ GATA3^+^. A similar subset of Th2-like Tregs has also been previously identified by mass cytometry (40). A fundamental role of GATA3 controlling Treg physiology has been shown in mice, where GATA3 was required for the maintenance of high levels of FOXP3 expression, and GATA3 promoted Tregs accumulation at inflamed sites (41). Thus, higher levels of effector memory Tregs expressing GATA3 in high-risk individuals might be explained by cell activation as result of the ongoing autoimmunity preceding disease onset. Sorted Tregs from the high-risk group had a similar expansion degree and suppressive capacity to the healthy group, confirming results from a previous study in children at high risk for T1D (38). As Tregs were sorted based only on the expression of CD25 and CD127, our findings raise the question whether subtle changes in specific Treg subpopulations, and not the whole Treg compartment are triggered as part of the regulatory process.

We were not able to detect Th1-like Tregs, in agreement with previous results from a study also defining Tregs by mass cytometry with a broad panel of antibodies (40). While we observed that some cells among CCR4^+^ GATA3^+^ Tregs were also CXCR3^+^, they were scarce and did not form a defined cluster. As we analyzed Tregs by the simultaneous expression of intracellular and extracellular markers at single cell level, methodological differences when defining Tregs may explain differences between our and previous findings. A study in humans defined populations of Th1, Th2, and Th17-like Tregs using mass cytometry, but key transcription factors like FOXP3, GATA3 and Tbet were not included (42). Another study used a combination of different approaches for Th1, Th2, and Th17 Treg definition, but Tregs were previously sorted and simultaneous expression of all the markers was not analyzed (43).

The potential role of NK cells in T1D has been investigated both in mice and humans (44–47), but the mechanisms of action and phenotypic characteristics in relation to the disease are still poorly understood. NKs have been long considered a homogenous population of innate lymphocytes, but multiple lineages of NK cells with unique phenotypic characteristics have been described (48). Lower frequency of NK cells has been detected at the onset of T1D using a limited phenotypic definition with CD16^+^CD56^+^ (41). Although phenotyping of NK cells in our study included lower number of markers than those used for the definition of T cells, a large number of clusters within NK cells was identified, highlighting the advantage of simultaneous multi-parametric analysis. Indeed, we found two NK subsets that were increased in high-risk individuals. They both expressed CXCR3, a chemokine receptor that is commonly expressed on cells during migration to inflamed areas (49, 50). Infiltration of NK cells to the islets has been reported in NOD mice (44), and islet inflammation mediated mainly by NK cells has also been reported in human T1D (47). It was interesting that one of the NK subsets that were increased in the high-risk group expressed CD11c, a marker mostly expressed by myeloid cells. It has been suggested that expression of CD11c on NK cells may represent an activation-induced change that was related to disease exacerbation in multiple sclerosis (51). Thus, it is tempting to speculate that higher levels of NK cells expressing this marker might reflect activation due to the ongoing autoimmune process. Interestingly, the increment in this subset was not accompanied by increased proportion of IFNγ-producing CD8+CD11c+ NK cells. Further functional studies on sorted cells from other cohorts may clarify whether these cells represent mainly a subset with lytic activity.

One of the main challenges when processing the large amounts of data generated with mass cytometry is the computational analysis. Boolean gating is limited by the number of comparisons and is not well-suited for analyzing a vast number of immune features within subsets. Dimensionality reduction approaches such as t-SNE are becoming the standard analysis for mass cytometry data, as they allow simultaneous profiling of high-dimensional datasets in an unbiased fashion. The analysis of our large dataset was performed using two different computational methods. First, a combination of t-SNE visualization with the Phenograph clustering algorithm was applied. This approach stratified the events plotted in two-dimensional maps into clusters, providing an insight of the complexity of the major immune populations and the phenotypic characteristics of all subsets. Second, Citrus algorithm was used to create a classification model able to identify stratifying immune features that best predict differences between high-risk individuals and healthy controls. Using this multidimensional single-cell approach, we were able to study differentiation, activation and functional markers in major cell types including T, B, NK, monocytes, and DC. One interesting finding was the high degree of heterogenicity observed within the subpopulations. For instance, we confirmed results from previous studies showing a large differentiation degree of naïve T cell subsets (21, 25), in contrast to the previous idea that these cells are a homogenous population. In addition, the combination of a broad range of markers revealed the presence of rare cell subsets like B cells expressing Tbet and CD11c, NK cells expressing CD8 and CD11c and FOXP3^+^ Tregs expressing GATA3. Comparison between high-risk and control individuals did not reveal further differences than those detected in Tregs and NK cells. Although the presence of multiple β-cell autoantibodies is a strong predictor of T1D, we observed no alterations within the B cell compartment in agreement with other studies (52, 53). The extensive number of markers in our panel made it possible to simultaneously phenotype several cell types, but deeper characterization of B cells, monocytes and DC might require the inclusion of additional markers able to further delineate subsets within them. The development of the CyTOF novel platform, along with improvements in reagent availability will offer that possibility in the near future. Our results in this study might constitute a starting point for designing panels that might identify novel populations, disease-associated immune signatures, and predictors for response to treatment.

The pre-diabetic phase preceding T1D onset is still poorly characterized, and so far, there are no good biomarkers for progression from autoantibody positivity to clinical diabetes. Indeed, many of the results from studies with high-risk individuals show rather an association of pre-diabetic features to glycemic status. For instance, we have shown in a previous study that serum miRNAs in autoantibody positive children appeared to reflect glycemic status (54). A recent study including autoantibody positive high-risk individuals described activated circulating follicular helper T cells appearing close to the clinical manifestation of T1D, and correlation of these cells with the activity of the disease was observed only in children with impaired glucose tolerance (53). High-risk individuals in the present study did not show impaired glucose tolerance at the time of sampling, suggesting that the changes observed were not the result of metabolic imbalance, and might be potential biomarkers predictive of T1D.

In conclusion, we show that subpopulations of Treg and NK cells were increased in individuals positive for multiple autoantibodies before disease onset. To our knowledge, these are the first results showing alterations of a subset of Tregs and NK cells in individuals with high risk for T1D. Using a multidimensional phenotyping and unbiased clustering approach we were able to identify cell populations expressing markers which are not often analyzed in a conventional flow cytometry panel. This approach made it possible to find differences that otherwise would not have been detected. Our data provides new insight into disease-related immune signatures.

## Ethics Statement

This study was carried out in accordance with the recommendations of The Research Ethics Committee of the Faculty of Health Sciences at Linköping University, Sweden with written informed consent from all subjects. All participating children and responsible guardians were thoroughly informed and provided a written informed consent in accordance with the Declaration of Helsinki. The protocol was approved by The Research Ethics Committee of the Faculty of Health Sciences at Linköping University.

## Author Contributions

HB, LÅ, and RC designed the experiments. HB and LÅ performed experiments. HB and MP developed and optimized mass cytometry panel. MP conjugated antibodies and acquired mass cytometry data. HB analyzed the data. HB and RC interpreted the results. HB and RC wrote the manuscript. LÅ, MP, and JL reviewed the manuscript. JL and RC conceived the study. All authors read and approved the final manuscript.

### Conflict of Interest Statement

The authors declare that the research was conducted in the absence of any commercial or financial relationships that could be construed as a potential conflict of interest. Alberto Sada Japp (ajapp@pennmedicine.upenn.edu) contributed to the review of Michael Betts.
