# Supplementary material for: Mass Cytometry Identifies Distinct Subsets of Regulatory T Cells and Natural Killer Cells Associated With High Risk for Type 1 Diabetes
Source: Front Immunol. 2019 May 3;10:982. doi: 10.3389/fimmu.2019.00982 (PMC6509181; doi:10.3389/fimmu.2019.00982)
Supplement: Supplementary file 1 [file Data_Sheet_1.PDF]

Supplementary Material

Mass cytometry identifies distinct subsets of regulatory T cells and Natural Killer cells associated with high risk for Type 1 diabetes

Hugo Barcenilla*, Linda Åkerman, Mikael Pihl, Johnny Ludvigsson, Rosaura Casas

*** Correspondence:** Hugo Barcenilla: hugo.barcenilla@liu.se

1 Supplementary Figures and Tables

1.1 Supplementary Figures (1-5)

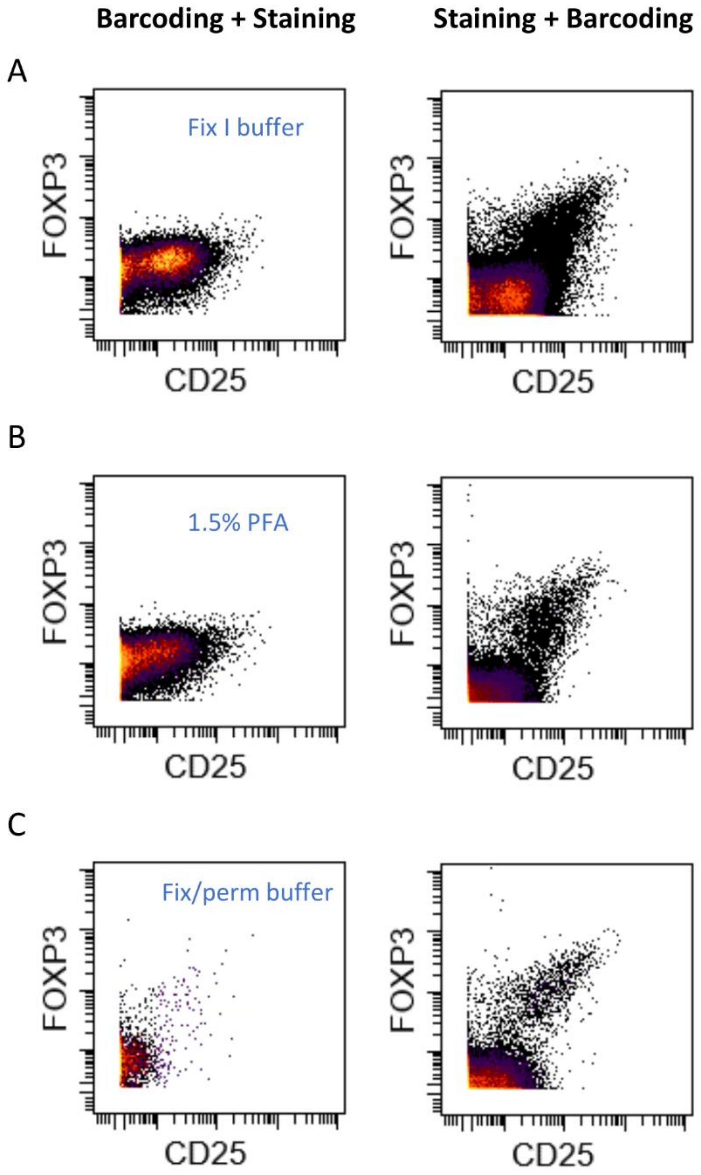

Supplementary Figure 1. Reduction in transcription factor staining after barcoding. Representative dot plots from three different parallel experiments, showing FOXP3 staining on gated CD4 T cells when barcoding was performed before (left) or after (right) cell staining. Different fixation buffers were tested for cell barcoding when performed before staining (Barcoding + Staining) **(A)** Maxpar® Fix I Buffer (Fix I buffer, fluidigm). **(B)** 1.5% paraformaldehyde (PFA). **(C)** FOXP3 fixation/permeabilization buffer (Fix/perm buffer, eBioscience). Barcoding after staining (Staining + Barcoding) was carried out on fixed cells using FOXP3 fixation/permeabilization buffer in the three experiments.

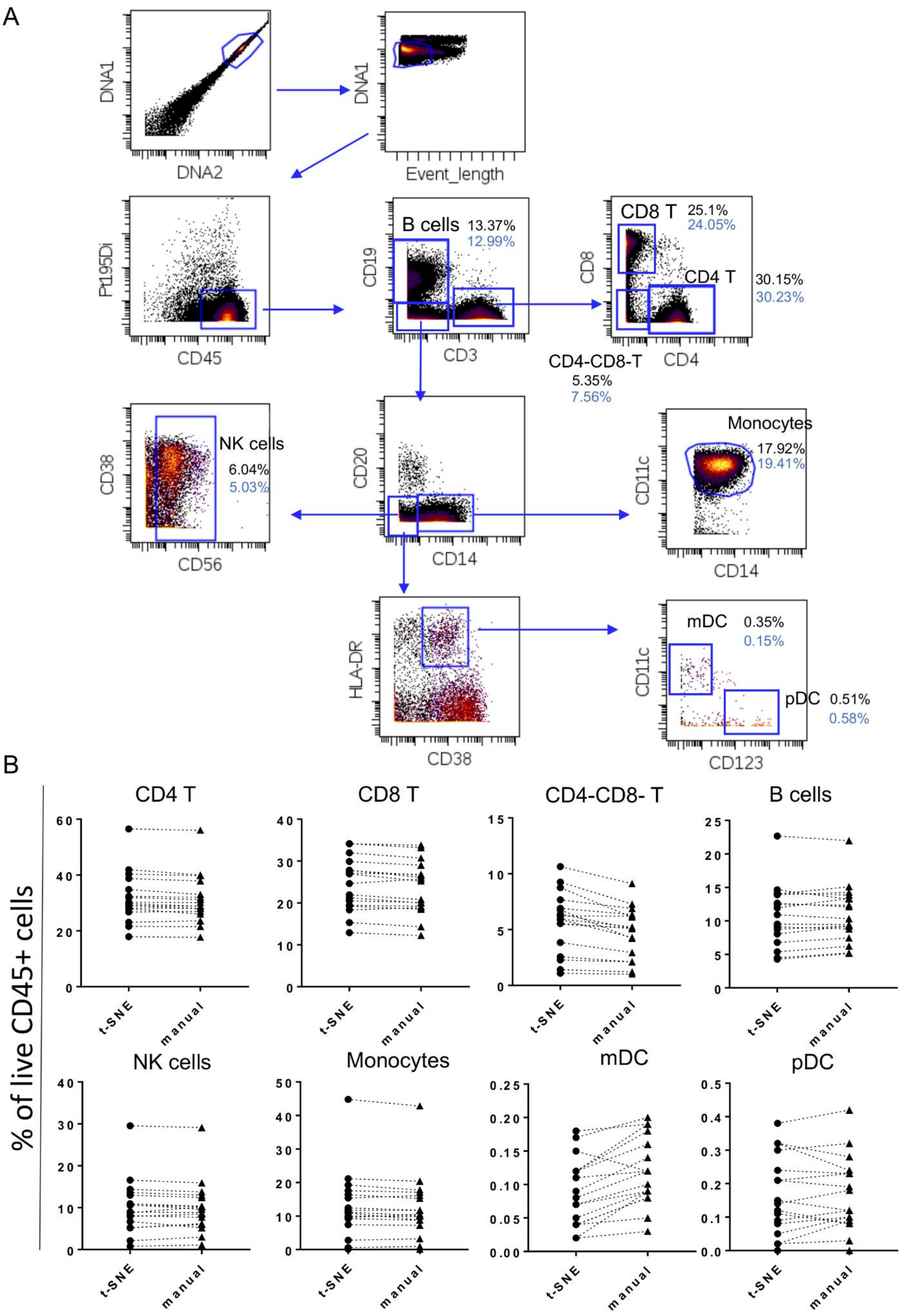
A
DNA1
DNA2
DNA1
Event_length
Pt195Di
CD45
B cells 13.37%
12.99%
CD19
CD3
CD8 T 25.1%
24.05%
CD8
CD4 T 30.15%
30.23%
CD4
CD4-CD8-T
5.35%
7.56%
NK cells
6.04%
5.03%
CD38
CD56
CD20
CD14
Monocytes
17.92%
19.41%
CD11c
CD14
HLA-DR
CD38
mDC 0.35%
0.15%
pDC 0.51%
0.58%
CD11c
CD123
B
% of live CD45+ cells
CD4 T
CD8 T
CD4-CD8- T
B cells
NK cells
Monocytes
mDC
pDC
t-SNE
manual

Supplementary Figure 2. Manual analysis of PBMC populations. **(A)** Gating strategy of a representative sample showing the percentages of the major immune populations within total CD45+ live cells. Blue numbers represent the percentages obtained by t-SNE analysis. **(B)** Cell frequencies obtained through t-SNE analysis and manual gating are shown for all samples. Dots represent individual samples. Differences between groups were tested using Wilcoxon signed rank test. No significant differences were found.

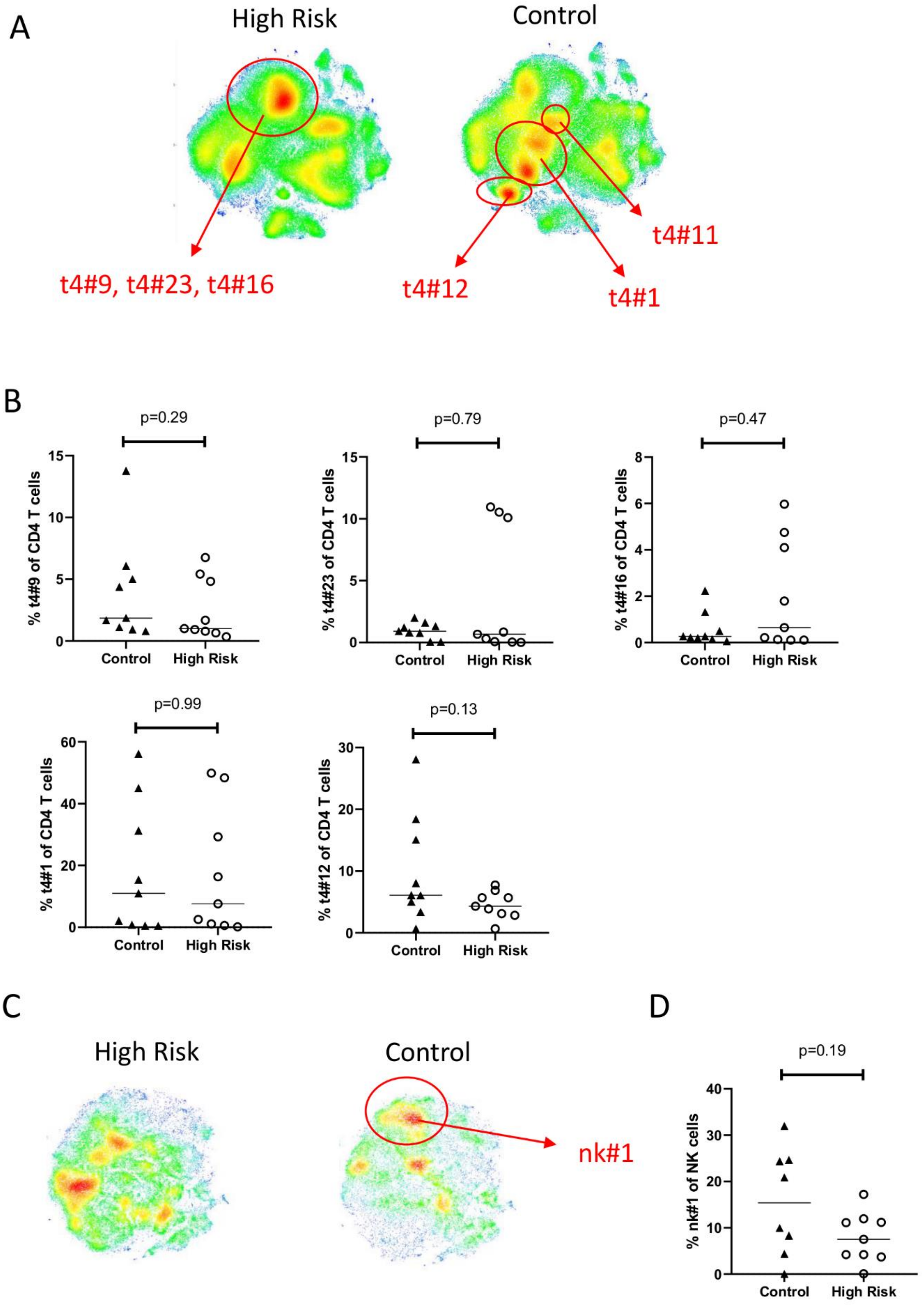

A
High Risk
Control
t4#9, t4#23, t4#16
t4#12
t4#1
t4#11
B
p=0.29
% t4#9 of CD4 T cells
Control
High Risk
p=0.79
% t4#23 of CD4 T cells
Control
High Risk
p=0.47
% t4#16 of CD4 T cells
Control
High Risk
p=0.99
% t4#1 of CD4 T cells
Control
High Risk
p=0.13
% t4#12 of CD4 T cells
Control
High Risk
C
High Risk
Control
nk#1
D
p=0.19
% nk#1 of NK cells
Control
High Risk

Supplementary figure 3. Analysis of areas of different cell density between high-risk and control groups. **(A)** CD4+ T cell density-based t-SNE maps indicating subsets within regions of different densities. **(B)** Frequencies of each subsets for all samples. The subset t4#11 expressed unspecific markers and was excluded from the study. **(C)** NK cell density-based t-SNE map indicating the subset within a region of different density. **(D)** Frequencies of the subset nk#1 for all samples. Dots represent individual samples. Differences between groups were tested using Wilcoxon signed rank test.

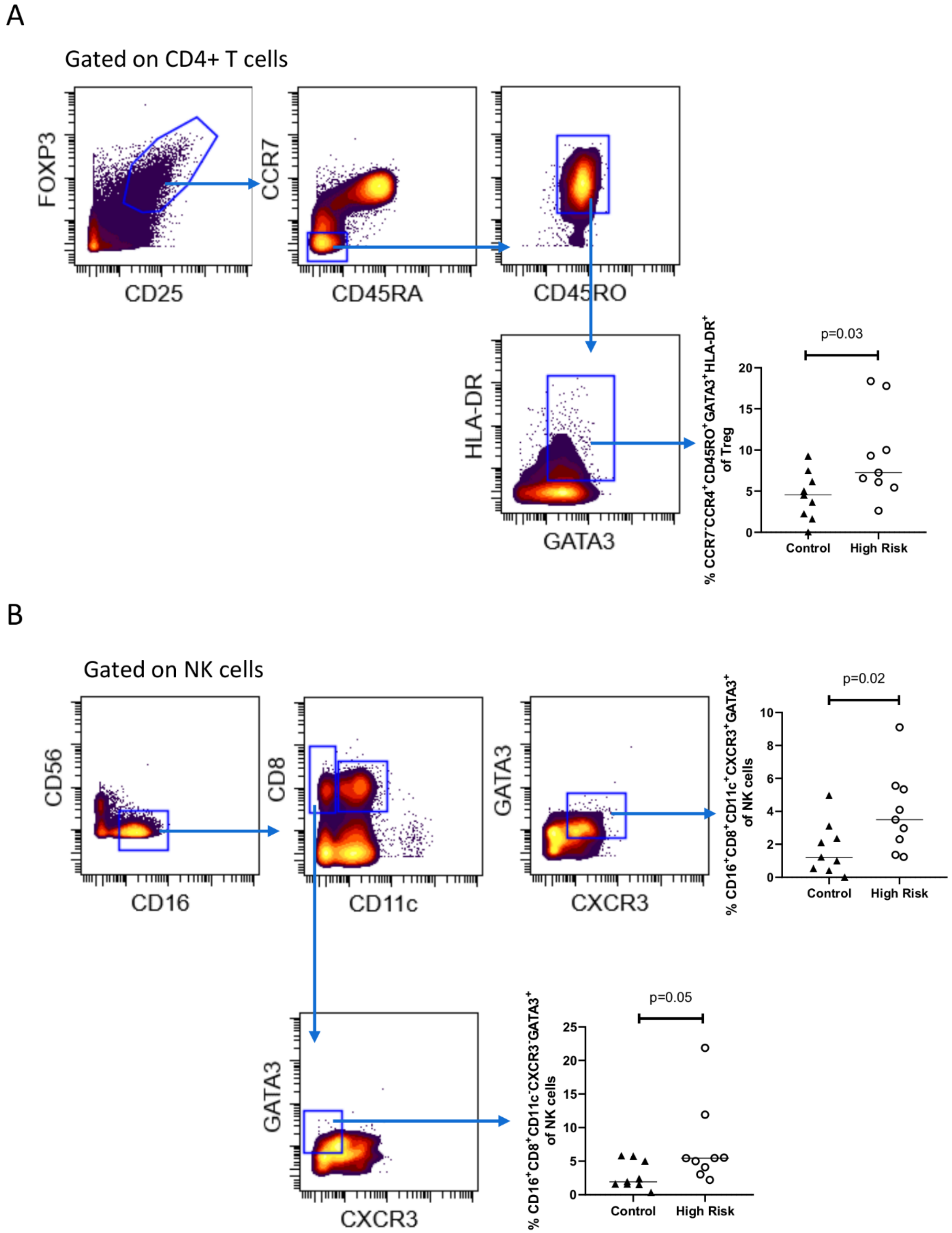
A
Gated on CD4+ T cells
FOXP3
CD25
CCR7
CD45RA
CD45RO
HLA-DR
GATA3
p=0.03
% CCR7-CCR4+CD45RO+GATA3+HLA-DR+ of Treg
20
15
10
5
0
Control
High Risk
B
Gated on NK cells
CD56
CD16
CD8
CD11c
GATA3
CXCR3
p=0.02
% CD16+CD8+CD11c+CXCR3+GATA3+ of NK cells
10
8
6
4
2
0
Control
High Risk
GATA3
CXCR3
p=0.05
% CD16+CD8+CD11c-CXCR3-GATA3+ of NK cells
25
20
15
10
5
0
Control
High Risk

Supplementary Figure 4. Manual analysis of the Treg and NK subsets increased in the high-risk group. **(A)** Gating strategy followed to define the Treg subset (t4#4) by manual analysis and the frequencies obtained through manual gating for all samples. **(B)** Gating strategy followed to define the NK subsets (nk#2 and nk#4) by manual analysis and the frequencies obtained through manual gating for all samples Dots represent individual samples. Differences between groups were tested using Wilcoxon signed rank test.

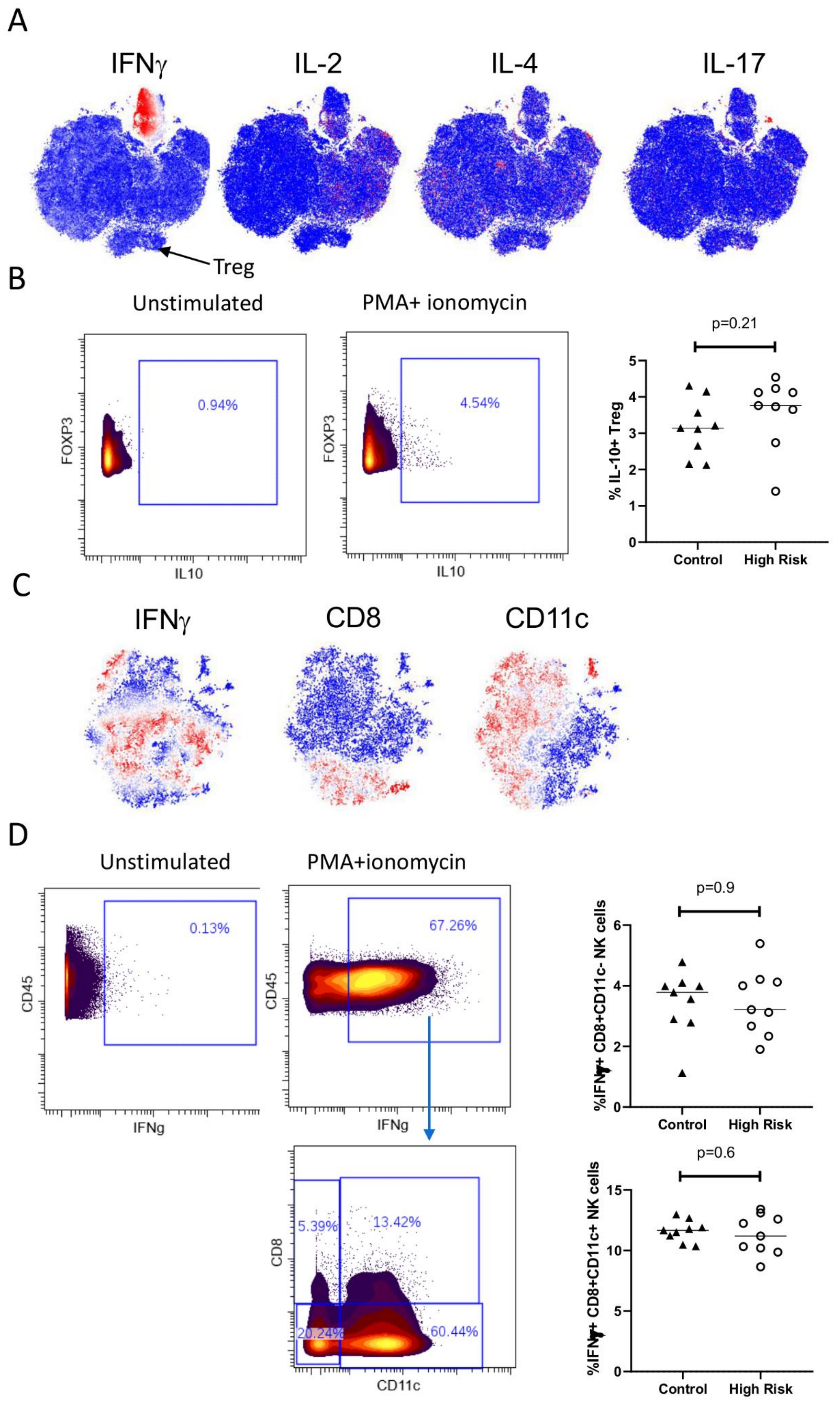

A
IFNγ
IL-2
IL-4
IL-17
Treg
B
Unstimulated
PMA+ ionomycin
0.94%
4.54%
FOXP3
IL10
p=0.21
% IL-10+ Treg
Control
High Risk
C
IFNγ
CD8
CD11c
D
Unstimulated
PMA+ionomycin
0.13%
67.26%
CD45
IFNg
5.39%
13.42%
20.24%
60.44%
CD8
CD11c
p=0.9
%IFNγ+ CD8+CD11c- NK cells
p=0.6
%IFNγ+ CD8+CD11c+ NK cells
Control
High Risk

Supplementary Figure 5. Analysis of the cytokine profile of Treg and NK cells. Samples of high-risk (n=9) and control (n=9) individuals were stimulated with PMA and ionomycin. **(A)** CD4+ T cell t-SNE plots of stimulated samples showing the expression of IFNg, IL-2, IL-4 and IL-17 outside the Treg area (black arrow). **(B)** IL-10+ Tregs were detected by manual gating in stimulated samples. Percentage of IL-10+ Tregs in high-risk and control individuals. **(C)** t-SNE plots showing the expression of IFNg, CD8 and CD11c within stimulated NK cells. **(D)** Manual gating of IFNg+ NK cells and percentage of CD8+CD11c+ and CD8+CD11c- NK cells in high-risk and control individuals. Dots represent individual samples. Differences between groups were tested using Wilcoxon signed rank test

1.2 Supplementary Tables (1-9)

Supplementary Table 1. Staining panel and details of antibodies. * conjugated in-house. EC: extracellular cocktail. IC: intracellular cocktail

Metal Tag	Target	Clone	Supplier	Usage
**^{102}Pd**	Barcode 1		Fluidigm	Barcoding
**^{104}Pd**	Barcode 2		Fluidigm	Barcoding
**^{105}Pd**	Barcode 3		Fluidigm	Barcoding
**^{106}Pd**	Barcode 4		Fluidigm	Barcoding
**^{108}Pd**	Barcode 5		Fluidigm	Barcoding
**^{110}Pd**	Barcode 6		Fluidigm	Barcoding
**^{89}Y**	CD45	HI30	Fluidigm	EC
**^{141}Pr**	CCR6	GO34E3	Fluidigm	EC
**^{142}Nd**	CD19	HIB19	Fluidigm	EC
**^{144}Nd**	IL4	MP4-25D2	Fluidigm	IC
**^{145}Nd**	CD4	RPA-T4	Fluidigm	Pre-staining
**^{147}Sm**	CD11c	Bu15	Fluidigm	EC
**^{148}Nd**	CD16	3G8	Fluidigm	EC
**^{149}Sm**	CD25	2A3	Fluidigm	EC
**^{150}Nd**	CD8a	RPA-T8	Biolegend *	EC
**^{151}Eu**	CD123	6H6	Fluidigm	EC
**^{152}Sm**	CD56	HCD56	Biolegend *	EC
**^{153}Eu**	CCR4	205410	Fluidigm	EC
**^{155}Gd**	CD27	L128	Fluidigm	EC
**^{156}Gd**	CD86	IT2.2	Fluidigm	EC
**^{158}Gd**	IL2	MQ1-17H12	Fluidigm	IC
**^{159}Tb**	CCR7	GO43H7	Fluidigm	EC
**^{160}Gd**	Tbet	4B10	Fluidigm	IC
**^{161}Dy**	CD69	FN50	Biolegend *	EC
**^{162}Dy**	FOXP3	PCH101	Fluidigm	IC
**^{163}Dy**	CXCR3	G025H7	Fluidigm	EC
**^{164}Dy**	IL17A	N49-653	Fluidigm	IC
**^{165}Ho**	CD45RO	UCHL1	Fluidigm	EC
**^{166}Er**	IL-10	JES3-9D7	Fluidigm	IC
**^{167}Er**	GATA3	TWAJ	Fluidigm	IC
**^{168}Er**	IFNg	B27	Fluidigm	IC
**^{169}Tm**	CD45RA	HI100	Fluidigm	EC
**^{170}Er**	CD3	UCHT1	Fluidigm	EC
**^{171}Yb**	CD20	2H7	Fluidigm	EC
**^{172}Yb**	CD38	HIT2	Fluidigm	EC
**^{174}Yb**	HLA-DR	L243	Fluidigm	EC
**^{175}Lu**	CD14	M5E2	Fluidigm	EC
**^{176}Yb**	CD127	A019D5	Fluidigm	EC
**$^{191/193}$Ir**	DNA		Fluidigm	DNA
**195 Pt**	Cisplatin		Fluidigm	live/dead

Supplementary Table 2. Characteristics of the CD4$^+$ T cell subsets (n=20) identified using t-SNE and Phenograph. Median intensity of each marker in each cluster was normalized in relation to the expression in total CD4$^+$ T cells using the arcsinh ratio function. Differential expression of each marker is presented as negative (-), low positive (+/-), positive (+), and high positive (++).

CLUSTER ID	CD27	CCR7	CD45RA	CD45RO	CD127	CD38	CCR6	CCR4	CXCR3	HLA-DR	CD69	CD25	FOXP3	TBET	GATA3	IL2	IFNγ	IL17A	IL10	IL4
t4#1	+	++	++	-	++	++	-	-	-	-	-	-	-	-	-	-	-	-	-	-
t4#2	+	++	+	-	++	++	-	-	-	+	-	-	-	-	-	-	-	-	-	-
t4#3	+	+	+	-	-	+	-	-	-	-	-	++	+	-	-	-	-	-	-	-
t4#4	+	-	-	++	-	-	+	++	+/-	+	-	++	+	-	+	-	-	-	-	-
t4#5	-	-	+/-	++	+/-	-	-	-	+	+/-	+/-	-	-	++	-	-	-	-	-	-
t4#6	-	-	-	++	+	-	+	+/-	-	+/-	+/-	-	-	-	-	+	+	+	-	-
t4#7	+	+	-	+	+	+	-	+	-	-	-	-	-	-	+	-	-	-	-	-
t4#8	+	++	+	-	+/-	+	-	-	-	-	-	-	-	-	-	-	-	-	-	-
t4#9	+	+	+	-	-	++	-	-	-	-	-	-	-	-	-	-	-	-	-	-
t4#10	+	-	-	+	-	-	-	+/-	-	-	-	-	-	-	-	+	-	-	-	-
t4#12	+	++	++	-	+	++	-	-	-	-	++	-	-	-	-	-	-	-	-	-
t4#13	-	-	-	+	-	-	-	-	+	-	-	-	-	+	-	+	+	-	-	-
t4#14	+	+/-	-	++	++	-	+	++	-	-	+/-	+	-	-	+	-	-	-	-	+
t4#15	+	+	+	-	+	+	+	+	-	-	-	-	-	-	-	-	-	-	-	-
t4#16	+/-	+	+	-	-	+	-	-	-	-	-	-	-	-	-	-	-	-	-	-
t4#17	+	+/-	-	++	++	-	+	+	+	-	+/-	-	-	+	+	-	-	-	-	-
t4#20	+	++	+	-	++	++	-	-	-	-	-	-	-	-	+	-	-	-	-	-
t4#21	+	+	+	-	-	++	-	-	-	-	-	-	-	-	-	+	-	-	-	-
t4#23	+	+	+	-	-	+	-	-	-	-	+/-	-	-	-	-	-	+	-	-	-
t4#24	-	-	-	+	+	-	+	++	-	-	-	+	-	-	+	+	-	-	-	-

Supplementary Table 3. Characteristics of the CD8^{+} T cell subsets (n=22) identified using t-SNE and Phenograph. Median intensity of each marker in each cluster was normalized in relation to the expression in total CD8^{+} T cells using the arcsinh ratio function. Differential expression of each marker is presented as negative (-), low positive (+/-), positive (+), and high positive (++).

CLUSTER ID	CD27	CCR7	CD45RA	CD45RO	CD127	CD38	CCR6	CCR4	CXCR3	CD16	CD56	CD11C	HLA-DR	CD69	CD25	FOXP3	TBET	GATA3	IL2	IFNγ	IL17A	IL10	IL4
**t8#1**	-	-	+	+	-	+	-	-	-	-	-	-	+/-	-	-	-	++	+/-	-	-	-	-	-
**t8#2**	-	-	++	-	-	-	-	-	-	-	-	+	-	-	-	-	+	-	-	+	-	-	-
**t8#3**	+	-	+	+	+	-	-	-	++	-	-	-	-	-	-	-	++	+	-	-	-	-	-
**t8#4**	-	-	+	-	-	+	-	-	-	-	-	-	+/-	+	-	-	++	+/-	-	-	-	-	-
**t8#5**	++	+	++	-	-	++	-	-	++	-	-	-	+/-	-	-	-	-	+	-	-	-	-	-
**t8#6**	-	-	+	+	-	-	+	++	++	-	+	-	-	-	-	-	++	-	-	+	-	-	-
**t8#7**	++	++	++	-	+	+	-	-	-	-	-	-	-	-	-	-	-	-	-	-	-	-	-
**t8#8**	++	+	++	-	-	+	-	-	-	-	-	-	-	-	-	-	-	-	-	-	-	-	-
**t8#9**	+	+	+	-	+	+	-	-	-	-	-	-	-	-	-	-	-	-	-	-	-	-	-
**t8#10**	++	-	+	-	+	-	-	-	++	-	-	-	-	-	-	-	+	+	-	-	-	-	-
**t8#11**	+	-	+	+	-	-	-	-	+	-	-	-	+/-	-	-	-	+	+/-	-	-	-	-	-
**t8#12**	++	+	++	-	+	+	-	-	+	-	-	-	-	-	-	-	-	-	-	-	-	-	-
**t8#13**	++	+	++	-	+	+	-	-	-	-	-	-	-	+	-	-	-	-	-	-	-	-	-
**t8#14**	++	+	++	-	+/-	+	-	-	+	-	-	-	-	-	-	-	-	-	-	-	-	-	-
**t8#16**	-	-	+	-	-	-	-	-	+	+/-	+	-	-	-	-	-	++	-	-	-	-	-	-
**t8#17**	+	-	+/-	+	+	-	+/-	-	+/-	-	-	-	-	+	-	-	+/-	+	-	-	-	-	-
**t8#18**	+	+/-	-	++	+	-	-	+	-	-	-	-	-	-	+/-	-	-	+	-	-	-	-	-
**t8#19**	++	-	+	-	-	-	-	-	+	-	-	-	-	-	-	-	-	-	-	-	-	-	-
**t8#20**	+	-	+/-	+	-	-	-	-	+	-	-	-	-	-	-	-	+/-	-	-	+	-	-	-
**t8#21**	++	+	+	-	+	+	-	-	+	++	-	-	-	-	-	-	-	-	-	-	+/-	-	-
**t8#23**	++	+	+	-	-	+	-	-	-	-	-	-	-	-	-	-	-	-	-	-	-	-	-
**t8#24**	-	-	+	-	-	-	-	-	-	-	-	-	-	-	-	-	+	-	-	+	-	-	-

Supplementary Table 4. Characteristics of the CD4$^-$CD8$^-$ T cell subsets (n=22) identified using t-SNE and Phenograph. Median intensity of each marker in each cluster was normalized in relation to the expression in total CD4$^-$CD8$^-$ T cells using the arcsinh ratio function. Differential expression of each marker is presented as negative (-), low positive (+/-), positive (+), and high positive (++).

CLUSTER ID	CD27	CCR7	CD45RA	CD45RO	CD127	CD38	CCR6	CCR4	CXCR3	CD16	CD56	CD11C	HLA-DR	CD69	CD25	FOXP3	TBET	GATA3	IL2	IFNγ	IL17A	IL10	IL4
dn#1	+	-	+	+	+	-	-	-	+	-	-	-	-	-	-	-	++	+	-	-	-	-	-
dn#2	+	-	+	+	+	-	-	-	+	-	-	-	-	+	-	-	++	+/-	-	-	-	-	-
dn#3	+	-	+	+	-	-	-	-	+	-	-	-	-	-	-	-	+	-	-	++	-	-	-
dn#4	-	-	++	-	-	++	-	-	-	-	-	+	-	-	-	-	++	-	-	+	-	-	-
dn#5	-	-	++	-	-	+	-	-	+	+/-	-	+	-	-	-	-	++	+/-	-	-	-	-	-
dn#6	-	-	++	-	-	+/-	-	-	-	+/-	-	-	+/-	+/-	-	-	++	+/-	-	-	-	-	-
dn#7	-	-	++	-	-	+/-	-	-	-	-	-	+	-	-	-	-	++	-	-	++	-	-	-
dn#8	-	+/-	+	+/-	-	+/-	+	+	++	-	-	-	+	-	-	-	+	-	-	-	-	-	-
dn#9	++	-	++	-	+/-	-	-	-	+	-	-	-	-	-	-	-	-	-	-	-	-	-	-
dn#10	+	-	+/-	+	+	-	+/-	-	+/-	-	-	-	-	+	-	-	+	+	-	-	-	-	-
dn#12	-	-	+/-	-	-	-	-	-	+/-	-	-	-	-	-	-	-	+	-	-	++	-	-	-
dn#13	++	+/-	+/-	+	-	+/-	-	+/-	-	-	-	-	-	-	+	+	-	-	-	-	-	-	-
dn#14	-	-	+/-	+	+	-	+/-	-	+	-	-	-	-	+/-	-	-	-	+	-	-	-	-	-
dn#15	++	+	++	-	+	-	-	-	+	-	-	-	-	-	-	-	-	-	-	-	-	-	-
dn#17	++	+	+	-	+	+	-	-	-	-	-	-	-	-	-	-	-	-	-	-	-	-	-
dn#18	+	-	+	+/-	+	-	-	-	++	-	-	-	-	-	-	-	++	+/-	-	-	-	-	-
dn#19	+	-	+/-	+	+	-	-	-	++	-	-	-	-	-	-	-	++	+/-	-	-	-	-	-
dn#20	++	+/-	++	-	+	-	-	-	+	-	-	-	-	+	-	-	-	-	-	-	-	-	-
dn#21	++	+	+	-	++	-	+	+	-	-	-	-	-	-	+	-	-	+	-	-	-	-	-
dn#22	+	-	-	+	+/-	-	-	-	-	-	-	-	-	-	-	-	-	-	-	-	-	-	-
dn#23	++	+	++	-	+/-	+	-	-	+	-	-	-	-	-	-	-	-	-	-	-	-	-	-
dn#24	+	-	+/-	+/-	-	-	-	-	+/-	-	-	-	-	+	-	-	+	-	-	++	-	-	-

Supplementary Table 5. Characteristics of the B cell subsets (n=17) identified using t-SNE and Phenograph. Median intensity of each marker in each cluster was normalized in relation to the expression in total B cells using the arcsinh ratio function. Differential expression of each marker is presented as negative (-), low positive (+/-), positive (+), and high positive (++).

CLUSTER ID	CD20	CD11C	CD123	HLA-DR	CD86	CD38	CD27	CCR7	CD45RA	CD45RO	CCR6	CCR4	CXCR3	CD127	CD69	CD25	FOXP3	TBET	GATA3	IL2	IFNγ	IL17A	IL10	IL4
b#1	+	-	-	+	-	+	+	+	+	-	+	-	-	-	-	+	-	-	-	-	-	-	-	-
b#2	+	-	-	+	+	++	++	-	+	-	-	+/-	-	-	-	-	+/-	-	-	-	-	-	-	-
b#3	-	-	-	+	-	+	-	+	+	-	+	-	-	-	-	-	-	-	-	-	-	-	-	-
b#4	-	-	-	+	-	+/-	-	-	+	-	-	-	-	-	-	-	-	-	-	-	-	-	-	-
b#5	+	-	-	+	-	+	-	+	+	-	++	-	-	+	-	-	-	-	-	-	-	-	-	-
b#6	+	-	+	+	-	+	-	+	+	-	+	+/-	-	-	-	-	-	-	-	-	-	-	-	-
b#7	+	-	-	+	-	+	-	-	+	-	+	-	-	-	-	-	-	-	-	-	-	-	-	-
b#8	+	-	-	+	-	+	-	+	+	-	+	-	-	-	+	-	-	-	-	-	-	-	-	-
b#11	+	-	-	+	-	+	-	+	+	-	+/-	-	-	-	+	-	-	-	-	-	-	-	-	-
b#12	+	-	-	+	-	+	-	+	+	-	-	-	-	-	-	-	-	-	-	-	-	-	-	-
b#14	-	-	-	+	-	-	+	+	++	-	+	-	+/-	-	-	+	-	-	-	-	-	-	-	-
b#17	+	-	-	+	-	+	-	+	+	-	+/-	-	-	-	-	-	-	-	-	-	-	-	-	-
b#18	+	-	-	+	-	+	-	+	+	-	+	-	-	-	-	-	-	-	-	-	-	-	-	-
b#19	+	-	-	+	-	+/-	+	+	+	-	+	-	+/-	-	+	+	-	-	-	-	-	-	-	-
b#20	+	+	-	+	+/-	+/-	-	+	+	-	+	-	+	-	-	-	-	+	-	-	-	-	-	-
b#21	+	-	-	+	-	-	-	-	+	-	+/-	-	-	-	-	-	-	-	-	-	-	-	-	-

Supplementary Table 6. Characteristics of the NK cell subsets (n=16) identified using t-SNE and Phenograph. Median intensity of each marker in each cluster was normalized in relation to the expression in total NK cells using the arcsinh ratio function. Differential expression of each marker is presented as negative (-), low positive (+/-), positive (+), and high positive (++).

CLUSTER ID	CD8	CD16	CD11C	HLA-DR	CD27	CCR7	CD45RA	CD45RO	CD127	CD38	CCR6	CCR4	CXCR3	CD69	CD25	FOXP3	TBET	GATA3	IL2	IFNγ	IL17A	IL10	IL4
nk#1	-	++	++	-	-	-	++	-	-	++	-	-	+/-	++	-	-	++	+	-	-	-	-	-
nk#2	+	+	-	-	-	-	++	-	-	++	-	-	-	++	-	-	++	+	-	-	-	-	-
nk#3	+/-	-	-	-	+	-	++	-	-	+	-	-	+	+	-	-	+	+	-	-	-	-	-
nk#4	+	+	+	-	-	-	++	-	-	++	-	-	+	-	-	-	++	+	-	-	-	-	-
nk#5	-	-	+	-	-	-	+	-	+	+	-	+/-	+/-	+	+	-	-	+	-	-	-	-	-
nk#7	-	++	++	-	-	-	++	-	-	++	-	-	+	-	-	-	++	+	-	-	-	-	-
nk#8	-	-	++	-	-	-	++	-	-	++	-	-	-	+	-	-	+	-	-	+	-	-	-
nk#10	+	++	++	-	-	-	++	-	-	++	-	-	+/-	++	-	-	++	+	-	-	-	-	-
nk#11	-	++	-	+/-	-	-	++	-	-	+	-	-	+	+/-	-	-	++	+	-	-	-	-	-
nk#12	-	+	-	-	-	-	++	-	-	++	-	-	+/-	++	-	-	++	+	-	-	-	-	-
nk#14	+	+	-	-	-	-	++	-	-	-	-	-	+/-	++	-	-	++	+	-	-	-	-	-
nk#16	-	+	-	-	-	-	++	-	-	+	-	-	+	-	-	-	++	+	-	-	-	-	-
nk#17	-	-	-	-	-	-	++	-	-	+	-	-	-	+/-	-	-	+	-	-	+	-	-	-
nk#18	+/-	-	++	+/-	+	-	+	-	-	++	-	-	+	-	-	-	++	++	-	-	-	-	-
nk#19	-	++	-	-	-	-	++	-	-	++	-	-	-	-	-	-	++	+	-	-	-	-	-
nk#21	+	-	++	-	-	-	++	-	-	++	-	-	-	+/-	-	-	+	-	-	+	-	-	-

Supplementary Table 7. Characteristics of the Monocyte subsets (n=18) identified using t-SNE and Phenograph. Median intensity of each marker in each cluster was normalized in relation to the expression in total Monocytes using the arcsinh ratio function. Differential expression of each marker is presented as negative (-), low positive (+/-), positive (+), and high positive (++).

CLUSTER ID	CD16	CD56	CD11C	CD123	HLA-DR	CD86	CD38	CD45RA	CD45RO	CD27	CCR7	CCR6	CCR4	CXCR3	CD127	CD69	CD25	FOXP3	TBET	GATA3	IL2	IFNγ	IL17A	IL10	IL4
mn#1	-	-	+	+/-	+	+	+	+/-	+	-	-	-	-	-	-	-	-	-	-	-	-	-	-	-	-
mn#2	++	-	+	+	++	++	-	++	-	-	-	-	+	+/-	-	+	-	-	-	-	-	-	-	-	-
mn#3	+	-	+	+	+	+	+	+	+	-	-	-	+	+/-	-	+	-	-	-	-	-	-	-	-	-
mn#4	-	-	+	+/-	+	-	+	+	+	-	-	-	+	+/-	-	+/-	-	-	-	-	-	-	-	-	-
mn#5	+	-	+	+	+	+	+	+	+	-	-	-	+	+	-	-	-	-	-	-	-	-	-	-	-
mn#6	+	-	+	+	+	+	+	+	+	-	-	-	+	+/-	-	+	-	-	-	-	-	-	-	-	-
mn#7	++	+/-	+	-	-	-	+	++	-	-	-	-	+/-	+	-	+	-	-	+	+	-	-	-	-	-
mn#8	-	-	+	+	+	+	+	+	+	-	-	-	+/-	-	+/-	-	+	-	-	-	-	-	-	-	-
mn#9	+	-	+	+	++	+	+	-	+	-	-	-	-	-	+/-	-	-	-	-	-	-	-	-	-	-
mn#10	-	-	+	+	++	-	+	-	+	-	-	-	-	-	-	-	-	-	-	-	-	-	-	-	-
mn#11	-	-	+	+	++	+	+	-	+	-	-	-	-	-	-	+	-	-	-	-	-	-	-	-	-
mn#12	+	-	+	+/-	+	+	+	+	+	-	-	-	+	+	-	+	-	-	-	-	-	-	-	-	+
mn#13	-	-	+	+	+	+	+/-	-	+/-	-	-	-	+/-	-	-	-	-	-	-	-	-	-	-	-	-
mn#14	-	-	+	+	+	+/-	+	+	+/-	-	-	+	++	++	-	-	-	-	-	-	-	-	-	-	-
mn#15	+/-	-	+	+	+	+	+	+	+	-	-	++	+	+	-	-	-	-	-	-	-	-	-	-	-
mn#16	-	-	+	+	++	+	+	+	+/-	-	-	-	-	-	+/-	-	-	-	-	-	-	-	-	-	-
mn#17	+/-	-	+	+	++	++	+	+	+/-	-	+	-	+	+/-	+	-	+	-	-	-	-	-	-	-	-
mn#18	-	-	+	-	++	-	+	-	+	-	-	-	-	-	-	-	-	-	-	-	-	-	-	-	-

Supplementary Table 8. Characteristics of the mDC subsets (n=9) identified using t-SNE and Phenograph. Median intensity of each marker in each cluster was normalized in relation to the expression in total mDCs using the arcsinh ratio function. Differential expression of each marker is presented as negative (-), low positive (+/-), positive (+), and high positive (++).

CLUSTER ID	CD16	CD56	CD11C	CD123	HLA-DR	CD86	CD38	CD45RA	CD45RO	CD27	CCR7	CCR6	CCR4	CXCR3	CD127	CD69	CD25	FOXP3	TBET	GATA3	IL2	IFNγ	IL17A	IL10	IL4
dc#1	-	-	++	-	+	+	+	+/-	++	-	-	-	-	-	-	-	-	-	-	-	-	-	-	-	-
dc#3	-	+	+	-	+	-	++	++	-	-	-	-	-	+	-	-	+	-	++	++	-	-	-	-	-
dc#4	-	-	++	-	++	++	++	+	+	-	+	-	+/-	+	+	-	-	-	-	-	-	-	-	-	-
dc#5	-	-	++	-	++	++	++	+	+	-	+	+/-	+/-	-	+	-	++	-	-	-	-	-	-	-	-
dc#6	+/-	-	++	-	+	++	+	+	+	-	-	-	+/-	-	-	-	-	-	-	-	-	-	-	-	-
dc#9	-	-	++	-	+	-	+	-	++	-	-	-	-	-	-	-	-	-	-	-	-	-	-	-	-
dc#10	-	-	++	-	+	+/-	+	+/-	+	-	-	-	-	-	-	-	++	-	-	-	-	-	-	-	-
dc#11	-	-	++	-	+	+	++	+	+/-	-	-	-	-	-	-	-	-	-	-	-	-	-	-	-	-
dc#12	-	-	++	-	+	-	+	+	+/-	-	-	+	++	++	-	-	-	-	-	-	-	-	-	-	-

Supplementary Table 9. Characteristics of the pDC subsets (n=8) identified using t-SNE and Phenograph. Median intensity of each marker in each cluster was normalized in relation to the expression in total pDCs using the arcsinh ratio function. Differential expression of each marker is presented as negative (-), low positive (+/-), positive (+), and high positive (++).

CLUSTER ID	CD16	CD56	CD11C	CD123	HLA-DR	CD86	CD38	CD45RA	CD45RO	CD27	CCR7	CCR6	CCR4	CXCR3	CD127	CD69	CD25	FOXP3	TBET	GATA3	IL2	IFNγ	IL17A	IL10	IL4
pdc#1	-	-	-	+	+	-	+	+	-	-	-	-	+/-	++	-	-	-	-	-	-	-	-	-	-	-
pdc#2	-	-	-	+	+	+	+	+	-	-	+/-	-	+	+	+	-	-	-	-	+/-	-	-	-	-	-
pdc#3	-	-	-	+	+	-	+	+	-	-	-	-	+/-	+/-	-	-	-	-	-	-	-	-	-	-	-
pdc#4	-	-	-	+	+	-	+	+	-	-	+	-	+	+/-	-	-	-	-	-	-	-	-	-	-	-
pdc#6	-	-	-	+	+	+	+	+	-	-	+	-	+/-	+/-	+	-	-	-	-	-	-	-	-	-	-
pdc#7	-	-	-	+	+	+/-	+	+	-	-	+	-	+/-	+/-	+/-	+	+	-	-	-	-	-	-	-	-
pdc#8	-	-	-	+	+	-	+	+	-	-	+/-	-	+/-	++	-	-	-	-	-	-	-	-	-	-	-
pdc#9	-	-	-	+	+	-	+	+	-	-	-	-	+	++	-	-	-	-	-	-	-	-	-	-	-
